# Structure of Benthic Microbial Communities in the Northeastern Part of the Barents Sea

**DOI:** 10.3390/microorganisms12020387

**Published:** 2024-02-15

**Authors:** Aleksandra R. Stroeva, Alexandra A. Klyukina, Olesya N. Vidishcheva, Elena N. Poludetkina, Marina A. Solovyeva, Vladislav O. Pyrkin, Liliya A. Gavirova, Nils-Kåre Birkeland, Grigorii G. Akhmanov, Elizaveta A. Bonch-Osmolovskaya, Alexander Y. Merkel

**Affiliations:** 1 Lomonosov Moscow State University, 119234 Moscow, Russia; 2 Winogradsky Institute of Microbiology, Research Center of Biotechnology, Russian Academy of Sciences, 119071 Moscow, Russia; 3 Department of Biological Sciences, University of Bergen, P.O. Box 7803, NO-5020 Bergen, Norway

**Keywords:** the Barents Sea, microbial ecology, microbial community structure, 16S rRNA profiling, benthic microbial communities, statistical analysis, correlation analysis

## Abstract

The Barents Sea shelf is one of the most economically promising regions in the Arctic in terms of its resources and geographic location. However, benthic microbial communities of the northeastern Barents Sea are still barely studied. Here, we present a detailed systematic description of the structures of microbial communities located in the sediments and bottom water of the northeastern Barents Sea based on 16S rRNA profiling and a qPCR assessment of the total prokaryotic abundance in 177 samples. Beta- and alpha-diversity analyses revealed a clear difference between the microbial communities of diverse sediment layers and bottom-water fractions. We identified 101 microbial taxa whose representatives had statistically reliable distribution patterns between these ecotopes. Analysis of the correlation between microbial community structure and geological data yielded a number of important results—correlations were found between the abundance of individual microbial taxa and bottom relief, thickness of marine sediments, presence of hydrotrolite interlayers, and the values of pH and Eh. We also demonstrated that a relatively high abundance of prokaryotes in sediments can be caused by the proliferation of *Deltaproteobacteria* representatives, in particular, sulfate and iron reducers.

## 1. Introduction

In aquatic habitats, benthic processes play a critical role in biogeochemical cycling [1]. Most of the water enters the Arctic Ocean through the Barents Sea [2]. We can assume that the benthic microbial communities of the Barents Sea play an important role in shaping the biogeochemical cycles of the entire Arctic Ocean. Moreover, the shelf of the Barents Sea is one of the most economically promising regions in the Arctic due to its resources and geographical location [3]. Several oil and gas fields, including one of the world’s largest, the Shtokman gas condensate field, were discovered in the southern Barents Sea [4,5]. However, the northeastern part of the Barents Sea is still relatively barely studied.

In the Cenozoic era, this region developed under the influence of several glaciations when ice sheets covered the entire Barents Sea shelf [6,7]. As a result of intense glacial erosion during the Cenozoic era, the Quaternary deposits lay unconformable on the Mesozoic series and are represented in the lower intervals by a complex of moraines that differ in age and genesis, mainly by dense, massive, almost impermeable clays with admixture of gravel material [8]. Glacial deposits seem to be a good lithological barrier for fluids migrating from the deeply buried sedimentary sequence to the sea bottom surface and are often covered with rather thin Upper Pleistocene glacial–marine and Holocene marine sediments. The modern bottom relief of the Barents Sea is formed by glacial, glacial–marine, and marine morphogenetic complexes of underwater hills and troughs and is complicated by local erosional and accumulative structures due to iceberg activity and fluid discharge processes [9,10].

In the bottom sediments of the Barents Sea, numerous interrelated processes take place (such as the deposition of organic and inorganic compounds, degradation of organic matter (OM) by microorganisms, dissolution of minerals, etc.) that are critical to the carbon cycle, as well as to the other nutrient cycles [11]. A clear stratification of microbial communities in sediments with depth was observed [11,12,13]. It was shown that microbial abundance in the Barents Sea decreases with sediment depth, with maximum microbial abundance found in the first 3 to 5 cm of sediment [11,14]. Data on the composition of microbial communities in Barents Sea sediments are fragmentary. The upper aerobic layer of sediments is dominated by groups of organotrophic microorganisms, often widely represented in the water column as well. The most abundant phyla are *Pseudomonadota*, *Bacteroidota*, *Actinomycetota*, *Verrucomicrobiota*, and *Desulfobacterota* [12,15,16,17,18].

The processes of migration and dispersion of liquid and gaseous hydrocarbons have been most actively studied in the Barents Sea water body [19]. In particular, a series of studies on pockmarks as the indicators of degassing processes have been published [20,21]. The release of methane from sediments is known to result in the formation of microbial communities that utilize it as a trophic source, and the composition of these communities can vary widely [22,23,24]. For the northern part of the Barents Sea, it was shown that rates of diverse microbially driven biogeochemical processes increased in the bottom sediments with a high methane concentration [18].

The aim of our work was a detailed systematic description of microbial community structures in the sediments and bottom water of the northeastern Barents Sea based on a rigorous statistical approach.

## 2. Materials and Methods

### 2.1. Study Area

**Figure 1:**
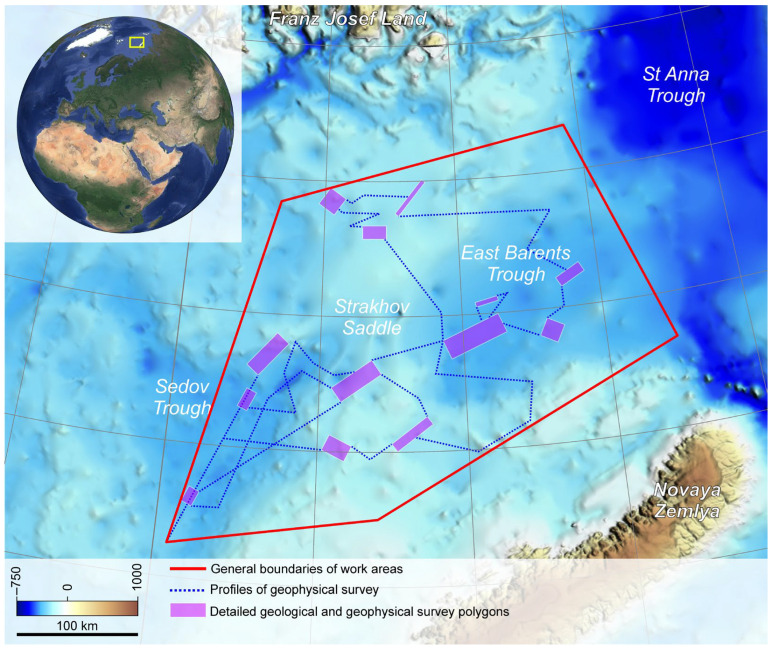
Investigation area map with the location of polygons indicating detailed geological and geophysical surveys and sites of bottom sampling.

This work was based on the data collected during the TTR-19 cruise (Training-through-Research (TTR) Program) in the summer of 2020 (RV “Akademik Nikolaj Strakhov”). It was a 30-day multidisciplinary research expedition to the northeastern Barents Sea covering a sector of approximately 80,000 km^2^ between Franz Josef Land and Novaya Zemlya, including the East Barents Trough, the eastern part of the Sedov Trough, and the Strakhov Saddle between them (Figure 1).

### 2.2. Sample Collection

The bottom sampling sites were selected based on multibeam and acoustic profiling data [9]. Bottom sediments were sampled with a gravity core (4 m long and about 800 kg in weight) with an inserted plastic liner. A total of 41 cores were collected.

For 16S rRNA gene-based profiling of microbial communities of the 36 uppermost layer samples (UL), 40 samples of sediments at a 5 cm depth below the bottom surface (S5 cm) and 27 samples of sediments at a 25 cm depth below the bottom surface (S25 cm) were used.

The bottom water samples from 37 sampling sites were obtained from the top of the gravity tubes, which were closed tightly when lifted. In order to concentrate the cells, the water was filtered consecutively through two types of filters: a Whatman^®^ (Maidstone, UK) Grade GF/C glass microfiber filter, 1.2 μm (prefiltration, PF), and a Durapore^®^ (Merck Millipore, Burlington, MA, USA) Membrane Filter, 0.22 µm (final filtration, F). At each sampling site, 0.5 to 3 L of bottom water was filtered. Further analytical procedures were performed for PF and F samples separately.

pH and Eh values were measured by the direct potentiometric method using an Expert–001 device (Electronpribor, Fryazino, Russia). Pyrolysis analysis was used for the identification of total organic carbon (TOC) content (Turbo Rock-Eval 6, Vinci Technologies, Nanterre, France). Methane content in the sediments was measured using the phase equilibrium degassing method on a Chromatec-Kristall 5000 gas chromatograph (Chromatec, Yoshkar-Ola, Russia).

### 2.3. DNA Extraction, 16S rRNA Gene-Based Profiling, and qPCR

DNA from sediment samples and filters was isolated using FastDNA Spin Kit for Soil (MP Biomedicals, Irvine, CA, USA) and TissueLyser LT (QIAGEN, Hilden, Germany), according to the manufacturer’s protocols. Library preparation of the V4 region of the 16S rRNA gene, sequencing, and analysis were performed as previously described [25]. Sequencing was carried out on a MiSeq system (Illumina, San Diego, CA, USA) using a reagent kit, which can read 150 nucleotides from each end. For each sample, this analysis was performed in two or more replicates. All stages of the analysis were accompanied by negative controls. All sequencing data were deposited into the NCBI SRA database under BioProject number PRJNA980746.

For qPCR-based total prokaryotes quantification, the same universal prokaryote primer system used for profiling was used: 515F (5′-GTGBCAGCMGCCGCGGTAA-3′) [26] and Pro-mod-805R (5′-GACTACNVGGGTMTCTAATCC-3′) [27]. For calibration curves, the genomic DNA of *Melioribacter roseus* P3M-2^T^ was used. Standards, samples, and negative controls were run in triplicate. qPCR analyses were carried out on a StepOnePlus Real-Time PCR System (Thermo Fisher Scientific, Waltham, MA, USA) using the qPCRmix-HS SYBR kit (Evrogen, Moscow, Russia).

### 2.4. Bioinformatics and Statistical Analysis

Initial sequence filtering, ASV table generation, and ASV classification were performed using Dadaist2 [28]. Filtration and normalization of the ASV table were performed using MicrobiomeAnalyst 2.0 [29]. ASVs that had a mean abundance value of less than 4 were filtered out. Filtering out low-represented phylotypes (ASVs) allows for the further analysis of only significant community components. ASVs that were close to constant throughout the samples were also filtered out (inter-quantile range of less than 5%). Filtering out phylotypes whose representation does not change significantly from sample to sample avoids possible contamination of the data. Total sum scaling was used for data normalization. Sequencing data need to be normalized due to differences in read depths.

The analysis of differences in community structures between different sample types (beta diversity analysis) was based on Jensen–Shannon Divergence and Principal Correspondence Analysis (PCoA) ordination, and the significance of differences between types of microbial communities was assessed by PERMANOVA. Alpha diversity analysis was based on the Shannon evenness index and the Chao1 richness estimator, and the significance of differences between types of microbial communities was assessed by ANOVA. The Chao1 richness estimator provides an estimate of the number of unique species in the community. The Shannon evenness index provides an estimate of community compositional complexity and richness. The statistical significance of the distribution of individual ASVs or phylogenetic groups they represent between different types of samples was assessed using a *t*-test/ANOVA. Visualization of the analysis results was carried out using MicrobiomeAnalyst 2.0 [29]. Correlation analysis was performed using Rhea [30].

## 3. Results

### 3.1. Characteristics of the Sampling Sites, the Bottom Water, and Upper Part of the Sediments

The depth of the water column within the studied area varied from 41 to 410 m. Different glacial landforms were detected on the sea bottom: megascale glacial lineations, moraine ridges of various origins, grounding zone wedges, subglacial tunnel valleys, hill-hole pairs, etc. The cored sediments were represented by Quaternary subglacial, glacial–marine, and marine deposits. Subglacial sediments were irregularly distributed over the study area. The thickness of this complex varied from 0–10 m (in the areas with flat bottom relief) to up to 100 m (on local positive relief structures or large hills). A complex of hardly subdivided glacial–marine and marine deposits covered the glacial deposits. The thickness of this complex generally varied from 0 to 6 m, gradually increasing from the Strakhov Saddle towards the deep parts of the Sedov Trough and the East Barents Trough. Variations in the thickness of the post-glacial sediments may indicate differences in the rate of sedimentation or hydrological regime in different parts of the studied area.

The bottom water showed pH values varying from 7.9 to 8.4, the salinity was around 34‰, and the temperature was from 0 to 3 °C. The bottom waters were characterized by positive values of redox potential (Eh)—from 206 to 250 mV.

The uppermost part of the bottom sediments (upper 25 cm) contained mainly silty clay that was gray or olive in color. The signs of sediment oxidation were often observed in the uppermost 10 cm of the section, which had a characteristic brownish color. This oxidized layer was often represented by water-saturated clay and silty clay, which became denser and more compact down the section. A distinctive feature of some stations was the presence of dense and clay-free sandy interlayers and lenses. Spots and interlayers of hydrotrolite were characteristic of most cores. Along the sampled sections, the pH values varied from 6.7 to 8.9; Eh varied from −147 in the deep sediment layers to 186 in the surface layers; and the TOC content varied from 0.64 to 2.05%. A study of the gas phase of the bottom sediments showed that shelf sediments of the northeastern part of the Barents Sea are characterized by low methane concentrations up to 28 ppm.

### 3.2. Microbial Community Composition

The microbial communities of the bottom water (prefilters and filters) and three different layers of sediments were studied using 16S rRNA gene-based NGS profiling and qPCR. In total, 177 samples were analyzed, and 1,196,095 sequences of the 16S rRNA gene V4 region were used in the analysis after all the initial steps of data processing. These sequences were merged into 5814 amplicon sequence variants (ASVs). For a meaningful analysis, the obtained data were filtered and normalized, and only 174 ASVs were used for further analysis (see Section 2).

#### 3.2.1. Total Numbers of Prokaryotes in the Bottom Water and Sediments of Barents Sea

The microbial communities of different sample types differed significantly in the total number of prokaryotes estimated by 16S rRNA gene-based qPCR. The average abundance of prokaryotes in PF samples was 6.4 × 10^5^ 16S rRNA gene copies calculated per 1 mL of seawater, standard deviation (SD) 2.2 × 10^5^, and in the F samples, the average abundance was 3.6 × 10^3^ 16S rRNA gene copies per 1 mL (SD 7.7 × 10^2^). The average number of prokaryotes in the uppermost sediment layer was three orders of magnitude higher: 3.7 × 10^8^ 16S rRNA gene copies per 1 cm^3^ of sediment (SD 9.4 × 10^7^). This quantity decreased sharply when moving deeper into the sediments, from 8.5 × 10^7^ (SD 2.8 × 10^7^) in the 5 cm layer below the surface (S5 cm) to 3.1 × 10^6^ (SD 3.3 × 10^5^) in the 25 cm layer below the surface (S25 cm). The abundance of prokaryotes fluctuated the most in the PF and the S5 cm samples and was the most stable in the S25 cm samples.

#### 3.2.2. Comparison of Microbial Communities of the Different Types of Samples

**Figure 2:**
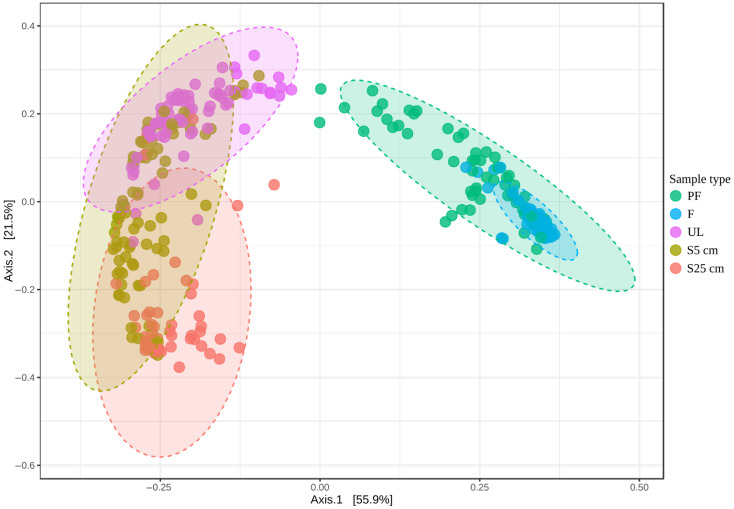
Analysis of differences in community structures between different sample types (beta diversity analysis). The visualization was based on counting the difference in the representation of microbial genera based on Jensen–Shannon Divergence and Principal Correspondence Analysis (PCoA) ordination. The significance of differences between microbial communities of different sample types based on the genus taxonomic level was assessed by PERMANOVA: F-value = 189.34; R^2^ = 0.67661; *p*-value = 0.001.

We analyzed the patterns of individual ASV distributions, as well as the phylogenetic groups they represented, in the samples of different types: prefilter (PF), filter (F), the uppermost layer of sediments (UL), 5 cm below the surface (S5 cm), and 25 cm below the surface (S25 cm). The diversity of microbial community structures in the different types of samples is illustrated by the results of the beta diversity analysis (Figure 2).

Aggregates of cells, possibly associated with organic particles, were concentrated on the prefilters, while single planktonic small-size cells were gathered on the filters. The high Eh of the bottom water indicates that these microorganisms are aerobes, while in cell aggregates gathered on the prefilters, the existence of anaerobic zones is not excluded. It can be seen from Figure 2 that sets of microorganisms gathered by the prefilters were represented by fairly diverse community structures, while those on the filters had much more specific composition, and both differed significantly from sediment communities. The UL and S25 cm samples represented compact sets of structures clearly divergent from each other (Figure 2). This discrepancy is probably caused by the difference in organic matter availability and composition in surface and deep layers, as well as by the oxygen availability. The communities of the S5 cm samples had common features both with the upper and the lower sediment layers.

**Figure 3:**
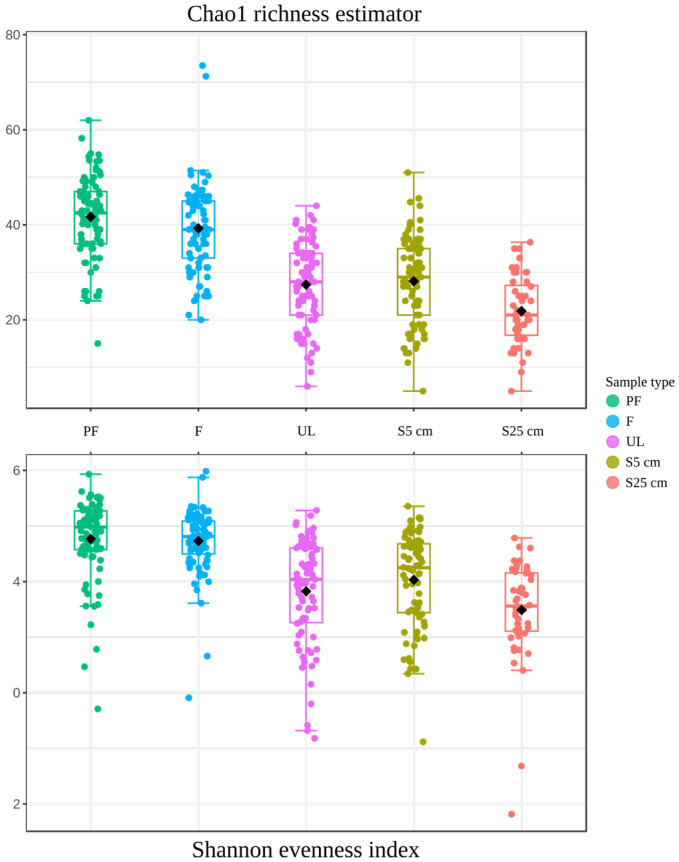
Visualization of the level of diversity within individual communities (Chao1 richness estimator) and complexity of their internal structure (Shannon evenness index). The significance of the discrepancy between microbial communities of different sample types was assessed by ANOVA: *p*-value = 1.4552^−38^; F-value = 59.733 for the Chao1 richness estimator and *p*-value = 3.9989^−23^; F-value = 32.535 for the Shannon evenness index.

**Figure 4:**
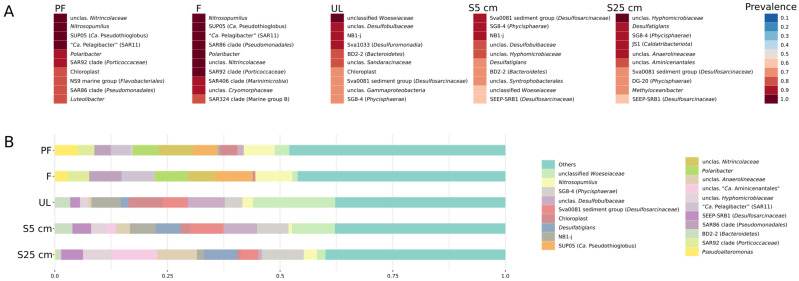
(**A**) Prevalence of the most consistent taxa in microbial communities of different sample types. The taxa with a relative abundance of at least 1% were taken into account. The presence of the 10 most constant taxa is shown. (**B**) Genus-level prokaryotic diversity in microbial communities of the different sample types based on 16S rRNA gene sequences (V4 region). Pooled normalized data across samples of the same type are shown. Group names are given according to SILVA SSU 138.1 [31].

The alpha diversity analysis shows the diversity value within individual communities (the Chao1 richness estimator) and the complexity of their internal structure (the Shannon evenness index). Both parameters reproduced approximately the same picture (Figure 3): the diversity and complexity of communities in the water samples were significantly higher than those of the sediment communities; among the latter microbial communities, the S25 cm samples were the least diverse and complex. The statistical significance of the results obtained was very high (*p*-value = 1.4552^−38^ for the Chao1 richness estimator and 3.9989^−23^ for the Shannon evenness index). Figure 4 shows the most consistent taxa in the microbial communities of the different sample types (Figure 4A) as well as genus-level prokaryotic diversity in the microbial communities of the different sample types (Figure 4B; Supplementary Table S1).

#### 3.2.3. Microorganisms Prevailing in the Bottom Water Samples

The patterns of microbial community structure from the filter (F) and prefilter (PF) samples were similar in several aspects (Figure 4; Supplementary Table S2). Among the groups significantly represented in both of these types of samples, there were representatives of the genus *Nitrosopumilus*. They were clearly dominant in the water samples (*p*-value 4.8 × 10^−56^, Supplementary Table S2) but were also present in all sample types including sediments (Supplementary Figure S1A). The average relative abundance of these microorganisms in the water microbial communities was 7–8% (Figure 4B; Supplementary Table S1). Bacteria of the genus *Polaribacter* accounted for 6–7% of the bottom water communities. They were detected only in water (Supplementary Figure S1B) and were also significantly represented in both types of water samples.

Microorganisms of SAR11 clade bacteria (*Alphaproteobacteria*) were represented by one ASV, which accounted for 4–7% of the bottom water communities and had 100% identity to “*Candidatus* Pelagibacter ubique”. It clearly predominated in the F samples (*p*-value 9.9 × 10^−12^) (Supplementary Table S2; Supplementary Figure S1C) and was absent in sediment samples. Among the groups lacking cultivated representatives, the SUP05 cluster was the most numerous and constant in the F samples. This cluster was also very homogeneous, being represented by only one ASV, which was highly related to “*Candidatus* Pseudothioglobus singularis” (99.53% identity). It was found strictly in water (Supplementary Figure S1D) where it accounted for 5–8% of the microbial communities. Its domination in the F samples (*p*-value 3.5 × 10^−10^) confirmed that it is a planktonic aerobic microorganism. Two other groups of quantitatively significant non-culturable microorganisms, the SAR86 and SAR92 clades, showed a highly significant prevalence in the F samples (*p*-value 2 × 10^−27^ and 3.3 × 10^−5^ respectively) (Supplementary Figure S1E,F). They accounted for 3 to 7% and 3 to 5% of prokaryotes in the water samples, respectively. Both groups were not homogeneous and were represented by a rather wide range of ASVs. Some other uncultured and cultured groups of bacteria showed an explicit tendency to be concentrated in the F samples: SAR406 (*p*-value 3.3 × 10^−18^), “*Candidatus* Actinomarina” (*p*-value 7.4 × 10^−14^), the NS4 marine group (*p*-value 3 × 10^−12^), the OM43 clade (*p*-value 8.9 × 10^−13^), *Nitrosomonas* (*p*-value 2 × 10^−12^), *Marinoscillum* (*p*-value 1.2 × 10^−11^), Clade IV (*p*-value 4.1 × 10^−8^), the NS5 marine group (*p*-value 1.3 × 10^−7^), and many others (Supplementary Table S2). It can be considered that all these groups are represented by planktonic aerobic small-size cells.

Another quantitatively significant group of microorganisms that was present only in the water samples was a heterogeneous group of several ASVs belonging to the *Nitrincolaceae* family (Supplementary Figure S1G). They accounted for 6 to 7% of the prokaryotes in the water samples and significantly prevailed in the PF samples (*p*-value 1.7 × 10^−5^), which indicated either their relatively large cell size, their association with particles, or cell aggregate formation. Some groups of microorganisms also showed strong prevalence in the PF samples. Among them were cultured organisms, e.g., *Paraglaciecola* (*p*-value 3.2 × 10^−12^) and *Luteolibacter* (*p*-value 2.5 × 10^−9^); some were uncultured and, in many cases, unclassified groups, such as unclassified genera of the families *Nitrincolaceae* and *Colwelliaceae* (*p*-value 1.7 × 10^−4^), the NS9 marine group (*p*-value 1.4 × 10^−4^), and others (see Supplementary Table S2).

#### 3.2.4. Microorganisms Prevailing in the Sediment Samples

In the sediment microbial communities, a clear change in the dominant groups from the communities of the uppermost layer of sediments (UL) to those at a depth of 25 cm (S25 cm) was observed. Unclassified *Woeseiaceae* was the most permanent and quantitatively significant group in the UL communities (Supplementary Figure S1H). The average abundance of this group decreased sharply with depth, from 18% in the UL samples to 2% in the S25 cm samples (*p*-value 6.9 × 10^−14^). In some cases, the percentage of representatives of this group in the communities was as high as 59%. In the samples studied, this group was not homogeneous and was represented by a large number of ASVs. Many of them had a relatively low level of 16S rRNA similarity to the type strain of *Woeseia oceani* (less than 94% similarity in the V4 hypervariable region). The genus *Desulfatiglans*—that of sulfate-reducing bacteria (SRB) and another constant and well-represented group in the sediment samples—exhibited the opposite trend: its relative abundance increased with depth from 1.5% in the UL samples to 7% in the S25 cm samples (Supplementary Figure S1I; *p*-value 2.6 × 10^−12^).

The majority of other quantitatively significant groups in the sediments were represented by uncultured microorganisms, such as the Sva0081 sediment group (family *Desulfosarcinaceae*), SEEP-SRB1, and SG8-4 (order *Sedimentisphaerales*, class *Phycisphaera*). They were detected in significant quantities in all three sediment layers and their abundance increased from the UL to deeper layers (Supplementary Table S2). The Sva0081 sediment group and SG8-4 were the most constant components of the sediment microbial community. The Sva0081 sediment group had the highest relative abundance in the S5 cm samples (8%) and the lowest in the UL samples (5%; Supplementary Figure S1J; *p*-value 8.5 × 10^−4^). The SG8-4 sediment group had the highest relative abundance in the S25 cm samples (8%) and the lowest in the UL samples (3%; Supplementary Figure S1K; *p*-value 6.7 × 10^−9^). Unfortunately, despite the fact that there are several publicly available MAGs of SG8-4 microorganisms, there is little to no information on their physiology. On the contrary, SEEP-SRB1 is a well-known group of SRB [32]. The relative number of the SEEP-SRB1 group representatives in the S5 cm and S25 cm samples was ~8%, whereas, in the UL samples, it was 4% (Supplementary Figure S1L; *p*-value 1.2 × 10^−4^).

Unclassified representatives of the *Desulfobulbaceae* family made up another numerous and constant group in the sediment samples. The share of the main ASV of the *Desulfobulbaceae* family was the highest in the surface layers of the sediments: from >1% in the S25 cm samples to 7 to 8% in S5 cm and UL (Supplementary Figure S1M; *p*-value 1.3 × 10^−13^).

The following taxa were also detected among the quantitatively significant groups of uncultured microorganisms whose populations increased towards the surface layers: unclassified representatives of the NB1-j phylum (6% in UL, 2% in S25 cm; Supplementary Figure S1N; *p*-value 5 × 10^−14^), Sva1033 (*Desulfuromonadales*) (4% in UL, >1% in S25 cm; Supplementary Figure S1O; *p*-value 1.6 × 10^−19^), and BD2-2 (*Bacteroidales*) (3% in UL, 1% in S25 cm; Supplementary Figure S1P; *p*-value 6.7 × 10^−6^).

There were also quantitatively significant groups of uncultured microorganisms whose populations increased towards the deeper layers (S25 cm). These are unclassified representatives of the order “*Candidatus* Aminicenantales” and the *Anaerolineaceae* and *Hyphomicrobiaceae* families. The order “*Candidatus* Aminicenantales” (<0.1% in UL, 9% in S25 cm; Supplementary Figure S1Q; *p*-value 7.1 × 10^−16^) was represented by a small group of ASVs that is quite distantly related (~89% similarity of V4 region of the 16S rRNA gene) to the known representatives of the genera “*Candidatus* Saccharicenans” and “*Candidatus* Aminicenans”. The *Anaerolineaceae* family (<1% in UL, 7% in S25 cm; Supplementary Figure S1R; *p*-value 3.5 × 10^−18^) was also represented by a group of ASVs only distantly related to the genus *Thermomarinilinea*. Finally, the *Hyphomicrobiaceae* family (1% in UL, 6% in S25 cm; Supplementary Figure S1S; *p*-value 1.4 × 10^−9^) was represented by two ASVs related to *Hyphomicrobium* sp. (~96% similarity).

The relative abundance of a rather broad set of minor phylogenetic groups was higher in the deep sediment layers (in S25 cm over the UL samples; Supplementary Table S2). In addition to all the above mentioned, the following groups should be noted—genus *Spirochaeta* (*p*-value 3.3 × 10^−13^), unclassified representatives of the *Calditrichaceae* family (*p*-value 9.2 × 10^−12^), unclassified representatives of the order-level lineages “*Candidatus* Aerophobales” (*p*-value 4.4 × 10^−9^), DG-20 (*Phycisphaerae*; *p*-value 3.6 × 10^−12^), FW22 (*Dehalococcoidia*; *p*-value 2.8 × 10^−7^), MSBL5 (*Dehalococcoidia*; *p*-value 2.4 × 10^−8^), S085 (*Dehalococcoidia*; *p*-value 8.1 × 10^−8^), vadinBA26 (*Dehalococcoidia*; *p*-value 1.3 × 10^−5^), unclassified representatives of the JS1 class-level lineage (*Caldatribacteriota*; *p*-value 3.8 × 10^−16^), and others.

### 3.3. Correlation between the Structure of Benthic Microbial Communities and Environmental Parameters

**Figure 5:**
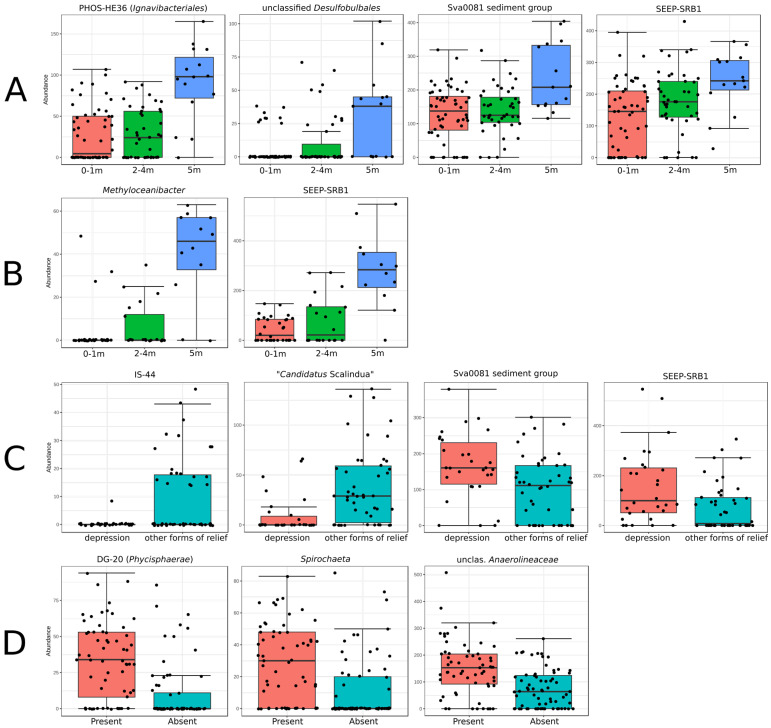
Distribution of ASV abundance of different groups in the normalized data of (**A**) the sediment samples (S25 cm and S5 cm) depending on the thickness of the marine sediment complex, (**B**) the UL samples depending on the thickness of the marine sediment complex, (**C**) the UL samples depending on the bottom morphology, and (**D**) the sediments samples (S25 cm and S5 cm) depending on the presence of hydrotroilite interlayers. Group names are given according to SILVA SSU 138.1 (Quast et al., 2013 [31]).

The thickness of sediments in the studied region of the Barents Sea varied from 0 to >100 m. The abundance of several groups increased at the sampling sites where the thickness of the marine sediment complex was above 5 m. In the S25 cm and S5 cm samples, these groups were PHOS-HE36 (*Ignavibacteriales*; *p*-value 3.1 × 10^−5^), unclassified *Desulfobulbales* (*p*-value 1.7 × 10^−4^), the Sva0081 sediment group (*p*-value 0.0017), and SEEP-SRB1 (*p*-value 0.002) (Figure 5A). In the UL samples, these groups were *Methyloceanibacter* (*p*-value 4.6 × 10^−10^) and SEEP-SRB1 (*p*-value 9.6 × 10^−9^) (Figure 5B).

The bottom morphology was found to cause a specific pattern in some microbial group distributions (Figure 5C). The prevalence of the Sva0081 sediment group (*p*-value 4.5 × 10^−4^) and SEEP-SRB1 (*p*-value 0.003) was characteristic for depressions, while “*Candidatus* Scalindua” (*p*-value 9.3 × 10^−5^) and IS-44 (*Nitrosomonadaceae*; *p*-value 1.8 × 10^−5^) were less common in such locations (Figure 5C).

It was also found that in the presence of hydrotroilite interlayers in the sediments, the relative abundance of DG-20 (*Phycisphaerae*; *p*-value 9.7 × 10^−8^), *Spirochaeta* (*p*-value 2.0 × 10^−6^), and unclassified representatives of *Anaerolineaceae* (*p*-value 7.7 × 10^−6^) (Figure 5D) was significantly higher than in locations without hydrotroilite interlayers.

There were also relatively weak correlations between the abundance of individual ASVs, as well as of the phylogenetic groups they represented, and the pH and Eh of the environment. All correlations found were for sediments only. The correlation coefficient did not exceed |0.64|. We considered only correlations with a coefficient (r) >|0.4| and a *p*-value of less than 0.005 and the condition that these thresholds were met when analyzing the two sequencing replicates separately (Supplementary Table S3). Two broadly represented ASVs showed a negative correlation with Eh. One of them belonged to unclassified *Desulfobulbaceae* (r −0.43; *p*-value 0.0007). This is the same ASV whose proportion of the sediment surface community was 7–8% and, in some cases, as high as 23% (Section 3.2.4). It is also worth noting the reliable negative correlation between Eh and the prevalence of representatives of the entire class *Desulfobacteria* (r −0.56; *p*-value 2.9 × 10^−10^). The other ASV that was negatively correlated with Eh belonged to unclassified *Hyphomicrobiaceae* (r −0.46; *p*-value 7.3 × 10^−6^). In addition, a weak negative correlation with Eh was shown by representatives of the family SG8-4 (r −0.4; *p*-value 2 × 10^−5^) and the order *Rhizobiales* (r −0.45; *p*-value 2.8 × 10^−6^). A positive weak correlation with Eh was shown by representatives of the class *Brocadiae* (r 0.59; *p*-value 0.0001) and phylum NB1-j (r −0.44; *p*-value 2.2 × 10^−5^).

Correlations between the prevailing microbial groups and pH values were also found only in the sediments. A positive correlation with pH was shown by the order *Polyangiales* (r 0.63; *p*-value 9.4 × 10^−6^) and the class *Ignavibacteria* (r 0.45; *p*-value 0.0008). A negative correlation was shown by the order *Nitrosococcales* (r −0.59; *p*-value 0.0009).

### 3.4. Correlations between Microbial Community Composition and Total Microbial Abundance

To identify the correlation between microbial abundance and microbial community composition, we compared the relative abundance of individual ASVs, as well as of the phylogenetic groups they represented, with the data on the total abundance of prokaryotes in a given sample obtained by qPCR. We considered only correlations with a coefficient (r) > |0.7|, a *p*-value less than 0.005, and the condition that these thresholds were met when analyzing the two sequencing replicates separately. Thus, we took into further consideration only very reliable patterns.

For several individual ASVs and several taxonomic groups, a reliable positive or negative correlation with the total abundance of prokaryotes in the community was shown (Supplementary Table S4). It is important to note that such correlations were found only in water and in the S25 cm samples, which were the samples with a relatively low total microbial abundance. In the S25 cm samples, the relative abundance of two SRB taxa had a high correlation coefficient with the increase in total microbial abundance: the genus *Desulfoconvexum* (r 0.94; *p*-value 4.5 × 10^−4^) and the family *Desulfobulbaceae* (r 0.91; *p*-value 1.7 × 10^−3^). A separate ASV belonging to *Sandaracinaceae* and representatives of the uncultured group Sva1033 (*Desulfuromonadales*) also had a high correlation coefficient with the increase in total microbial abundance in the sediment samples (r 0.92 and *p*-value 1.4 × 10^−3^; r 0.93 and *p*-value 7.3 × 10^−4^, respectively). A larger number of the correlations identified in the S25 cm samples were negative, as their relative abundance increased with the decrease in the total number of prokaryotes in the samples. Representatives of the class *Dehalococcoidia* had the most reliable negative correlation (r −0.96 and *p*-value 1.4 × 10^−4^). Similar results were obtained for representatives of the phyla *Chloroflexota* and *Acidobacteriota* (r −0.96 and *p*-value 1.9 × 10^−4^, r −0.95 and *p*-value 3.6 × 10^−4^, respectively). Representatives of the following groups also showed a negative correlation—WCHB1-81 (*Actinomycetota*; r −0.91 and *p*-value 1.8 × 10^−3^), *Lokiarchaeia* (r −0.91 and *p*-value 1.7 × 10^−3^), and *Desulfatiglandales* (r −0.93 and *p*-value 9.1 × 10^−4^). This indicates that these microorganisms exist in the S25 cm layer as a permanent background.

In the water samples, only a few negative correlations were seen (Supplementary Table S4). In the prefilter samples, the lower was the total number of prokaryotes, the greater was the relative representation of the *Flavobacteriales* and *Rhodobacterales* orders (r −0.85 and *p*-value 5.8 × 10^−5^, r −0.97 and *p*-value 2.2 × 10^−9^, respectively). In the F samples, the same tendency was shown by phylotypes of “*Candidatus* Pseudothioglobus singularis” (r −0.79 and *p*-value 5.1 × 10^−4^), *Thiomicrospirales* (r −0.78 and *p*-value 5.5 × 10^−4^), and the SAR406 clade (r −0.88 and *p*-value 1.2 × 10^−5^).

## 4. Discussion

Microbial communities of the world’s oceans play an important role in the cycles of biogenic elements on the earth and in maintaining biosphere stability. Therefore, due to the development of molecular methods for studying microbial communities, a large number of studies on the diversity of microorganisms in the marine habitats of our planet have appeared, including both 16S rRNA profiling and metagenomic analyses of different marine habitats [33,34,35,36,37]. Among them are many that focus on microbial communities of the Arctic regions and their spatial and seasonal changes. The majority of such studies in Arctic regions were devoted to planktonic microbial communities, mainly focusing on the upper water layers [38,39,40,41,42]. A smaller number of studies have looked at bottom sediments from the Arctic region, usually on a few (2 to 10) samples and predominantly their surface portion, no deeper than 10 cm [11]. In an interesting work describing the sediment communities of the Barents Sea [18], seven samples were studied, of which five were of surface horizons, one from a 6–7 cm depth, and only one from a 16–19 cm depth. As far as we know, nobody ever studied the composition of bottom-water microbial communities in the northeastern part of the Barents Sea.

The aim of the present work was to compare the diversity of microorganisms in the bottom water and different sediment layers of the northeastern part of the Barents Sea and to identify the available correlations between the composition of microbial communities and environmental parameters. The reliability of the obtained results was determined by analysis of a large number of samples and a rigorous statistical approach.

In all locations, we studied the aphotic zone of the Barents Sea. The source of energy substrates in bottom sediments and, partly, in bottom water is insoluble organic matter of plant and animal origin accumulating on the sea bottom. Partial decomposition of organic matter occurs already during its passage through the water column, and the greater the depth, the more of labile biomolecules will be hydrolyzed already in the water column. Bottom sediments also select organic molecules according to their degradability: the most accessible polymers are used in the upper layers, while those whose decomposition proceeds at a slower rate than sediment accumulation fall into the lower layers. Decomposition products of polymers are partly assimilated by hydrolytic microorganisms or other community members and partly diffuse into upper layers and into water; the most active diffusion of low-molecular organic compounds into water takes place in the upper layers of sediments. In our work, we identified 51 groups of microorganisms whose distribution between different sediment layers had a statistically significant pattern (Supplementary Table S2). In addition, the different sediment layers also differ significantly in total prokaryote abundance. For the top part of the Barents Sea sediments, a rough estimation could be made on the number of prokaryotes decreasing by an order of magnitude with every 10 cm of depth—from 4 × 10^8^ in uppermost layers to 3 × 10^6^ in sediments at the 25 cm depth below the bottom surface. These data correlate well with previously published data [11]. There are several quantitatively significant groups whose distribution clearly increased in the uppermost layer of sediments: unclassified (unclas.) *Woeseiaceae*, Sva1033 (*Desulfuromonadales*), unclas. *Desulfobulbaceae*, BD2-2, and NB1-j. To date, only a single representative of the family *Woeseiaceae* has been described—*Woeseia oceani*—a heterotrophic facultatively anaerobic gammaproteobacterium [43]. In our surface samples, the population of *Woesiaceae* was not homogeneous, indicating that the uppermost layer of Arctic marine sediments may be the source of new psychrophilic isolates of the family *Woeseiaceae*—a group that has the potential for a broad range of energy-yielding metabolisms [44]. For the Sva1033 group, it was previously predicted that its members were capable of dissimilatory iron reduction in cold marine sediments [45]. Together with other representatives of *Deltaproteobacteria*, including the above-mentioned unclas. *Desulfobulbaceae*, this group showed a high correlation coefficient with total microbial abundance. Thus, a relatively high abundance of prokaryotes in sediments can be caused by the development of representatives of *Deltaproteobacteria*, in particular, sulfate reducers and iron reducers.

There are also several groups whose prevalence increases in deeper sediment layers. The most quantitatively significant among them are unclas. *Hyphomicrobiaceae*, unclas. *Anaerolineaceae*, unclas. “*Ca.* Aminicenantales”, the Sva0081 sediment group, SG8-4, SEEP-SRB1, and the genus *Desulfatiglans*. These results indicate that representatives of these groups are anaerobes and can be adapted to the degradation of highly resistant and rarefied organic matter (OM). Some of these results were confirmed by analyzing the correlation between the relative abundance of microorganisms and the value of Eh. While an increase in the number of SRBs with decreasing Eh is predictable, the corresponding increase in *Hyphomicrobiaceae* indicates their preference for anaerobic growth. Despite the fact that most *Hyphomicrobiaceae* are aerobic chemoheterotrophs, a few representatives can grow anaerobically by denitrification or mixed-acid fermentation, which may explain their preference for reduced conditions. 

The abundance of the Sva0081 sediment group (*Desulfosarcinaceae*) and SEEP-SRB1 increased within layers where the thickness of the marine sediment complex was above 5 m. The dependence of microbial community structure on sediment thickness can be explained in two ways. Firstly, thick marine sediments may indicate a high sedimentation rate in a particular location. The groups of microorganisms whose relative abundance reliably increased in such locations were probably participating in intensive processes of OM decomposition. Secondly, the influence of thick marine sediments on microbial community composition can be explained by the inflow of low-molecular-weight complex OM degradation products from the lower into the upper sediment layers. It was previously assumed that Sva0081 is a group of SRB that is capable of acting as an important sink for H_2_ and acetate in marine sediments [46,47]. The SEEP-SRB1 group was originally described as an SRB partner of methanotrophic archaea ANME-2 [32]. These microorganisms are usually dominant in methane-rich sediments [48]. However, in our samples, we did not detect any ANME-2 sequences, which correlates with the low methane concentration in the sediments studied and suggests a different environmental function of the SEEP-SRB1 group in our samples. The abundance of Sva0081 and SEEP-SRB1 also increased in depressions versus other bottom reliefs such as hills, flats, and slopes. Depressions are supposed to accumulate more sediment than locations with a positive relief. These data also confirm the active participation of representatives of these SRB groups in the decomposition of incoming OM. 

Unclas. *Anaerolineaceae* was among the groups whose prevalence depended on the presence of hydrotrolite interlayers in the sediments. Hydrotrolite occurs in sediments when iron hydroxides interact with free hydrogen sulfide, which is a metabolic product of SRB. This mineral is widespread in silty and clayey reduced and organic-rich sediments. The prevalence of unclas. *Anaerolineaceae* and other groups of microorganisms in the sediments with hydrotrolite interlayers indicate that their representatives coexist with or participate in the processes of sulfate-dependent OM decomposition. Unclas. *Anaerolineaceae* was represented by a group of ASVs that were frequently detected in sediments associated with methane seeps (e.g., [49]) where sulfate-dependent OM decomposition is usually very active. The role of this group of microorganisms in these processes remains to be discovered. Another dominant group in deeper sediments was SG8-4, representing planktomycetes of the class *Phycisphaera*, belonging to the order *Sedimentimentisphaerales*. In the sediments of the Barents Sea, the SG8-4 group preferred anaerobic sediment horizons (it negatively correlated with Eh values) and probably shared the ability of other *Sedimentimentisphaerales* to anaerobically decompose simple and complex sugars [50,51].

Contrary to the existing notion that the same groups of microorganisms inhabit the bottom water and the upper sediment layer, we were able to show that in the samples we studied these types of communities barely overlapped (Figure 2). We identified 40 groups of microorganisms the distribution of which was statistically significantly higher in water than in sediments (Supplementary Table S2). The most quantitatively significant among them are *Nitrosopumilus*, “*Ca.* Pseudothioglobus”, *Polaribacter*, “*Ca.* Pelagibacter”, unclas. *Nitrincolaceae*, and the SAR86 clade. The genus *Nitrosopumilus* represents ammonia-oxidizing archaea that are ubiquitous and abundant in marine environments, commonly in the near-bottom water column [52] where they oxidize ammonium formed in sediments during the decomposition of protein substrates and diffused into the upper sediment layers and bottom water. Among the abovementioned groups, only *Nitrosopumilus* and *Polaribacter* did not show an explicit tendency to be enriched in one of the two cell fractions: those that were concentrated on the prefilter and those that were concentrated on the filter. The genus *Polaribacter* is a well-known cultivated bacterial group, a constant and abundant component of bottom-water microbial communities. At present, it includes 29 validly described species. They are heterotrophic, aerobic, psychrophilic, or mesophilic bacteria that are widely distributed in marine environments. Most of the other abovementioned groups showed a clear tendency to be concentrated on the filter. “*Ca.* Pseudothioglobus singularis” was one of the most numerous and constant groups in the filter samples. It has an organoheterotrophic aerobic lifestyle and does not oxidize sulfur, in contrast to another member of the SUP05 cluster, “*Ca.* Thioglobus autotrophicus” [53,54]. In our study, the phylotypes of this group showed a negative correlation with the total abundance of prokaryotes. This indicates that these microorganisms exist in the bottom water on a permanent basis. Another widely represented group of microorganisms in the filter samples were the representatives of “*Ca.* Pelagibacter” (SAR11), which are ubiquitous in marine environments. Cells of these microorganisms were found to be among the smallest free-living cells known (approximately 0.01 μm^3^) [55]. They are aerobic oligotrophs that can metabolize a wide variety of volatile and methylated compounds [56]. It should be emphasized that “*Ca.* Pelagibacter” was represented by a single ASV, i.e., it consisted of a single strain belonging to “*Ca.* Pelagibacter ubique”, which is widely distributed throughout the world’s oceans and apparently has some capacities that ensure its environmental success. Numerous other groups of bacteria prevailing in the filter samples are also mentioned in Supplementary Table S2. While for a number of these organisms, it is already known that they have very small cells (e.g., “*Ca.* Actinomarina” [57]) or have aerobic lifestyles (e.g., SAR406 [58]), for the other ones, this indication may become an important step in describing these groups’ physiology. Decomposition of large organic molecules can also take place in the water column, for example, in aggregates associated with particles, which, in the case of our studies, were trapped on the prefilter. Among the abovementioned most quantitatively significant groups, the only one that showed a tendency to be enriched on the prefilter was a heterogeneous group of ASVs belonging to the *Nitrincolaceae* family. It was reported that unclassified *Nitrincolaceae* were found in phytoplankton blooms in Antarctica and the Arctic and that the representatives of this family possessed diverse carbon utilization metabolic pathways and were able to respond rapidly to increased organic carbon availability [41]. However, in our study, we found no positive correlations between the relative abundance of phylotypes of this group and total microbial abundance. 

Based on the analysis of 177 samples, we were able to provide a systematic and detailed description of the benthic microbial communities of the northeastern Barents Sea. We identified 101 microbial taxa whose representatives had statistically reliable distribution patterns between the microbial communities of sediments and bottom water, as well as between the microbial communities of different sediment layers and different bottom water fractions. For many of the uncultured members of these groups, these data would help to reconstruct their physiology.

## Data Availability

The obtained 16S rRNA gene sequences were deposited in the NCBI Sequence Read Archive (SRA) and are available via the BioProject PRJNA980746. Data are contained within the article and Supplementary Materials.

## Supplementary Materials

The following supporting information can be downloaded at: https://www.mdpi.com/article/10.3390/microorganisms12020387/s1, Figure S1: Distribution of the ASV abundance of different groups in the normalized data for each sample type [31]; Table S1: List of microbial taxa whose representatives had statistically reliable distribution patterns between the studied ecotopes; Table S2: Relative abundance of the most numerous genus-level taxa across sample types [31]; Table S3: Correlation between the abundance of ASV or taxonomic groups and the pH and Eh of the environment; Table S4: correlation between the total abundance of prokaryotes in the community and the relative abundance of the ASVs and taxonomic groups of microorganisms.
