# Supplementary material for: Structure of Benthic Microbial Communities in the Northeastern Part of the Barents Sea
Source: Microorganisms. 2024 Feb 15;12(2):387. doi: 10.3390/microorganisms12020387 (PMC10892650; doi:10.3390/microorganisms12020387)
Supplement: Supplementary file 1 [file microorganisms-12-00387-s001.zip › Suppl.Fig.S1.docx]

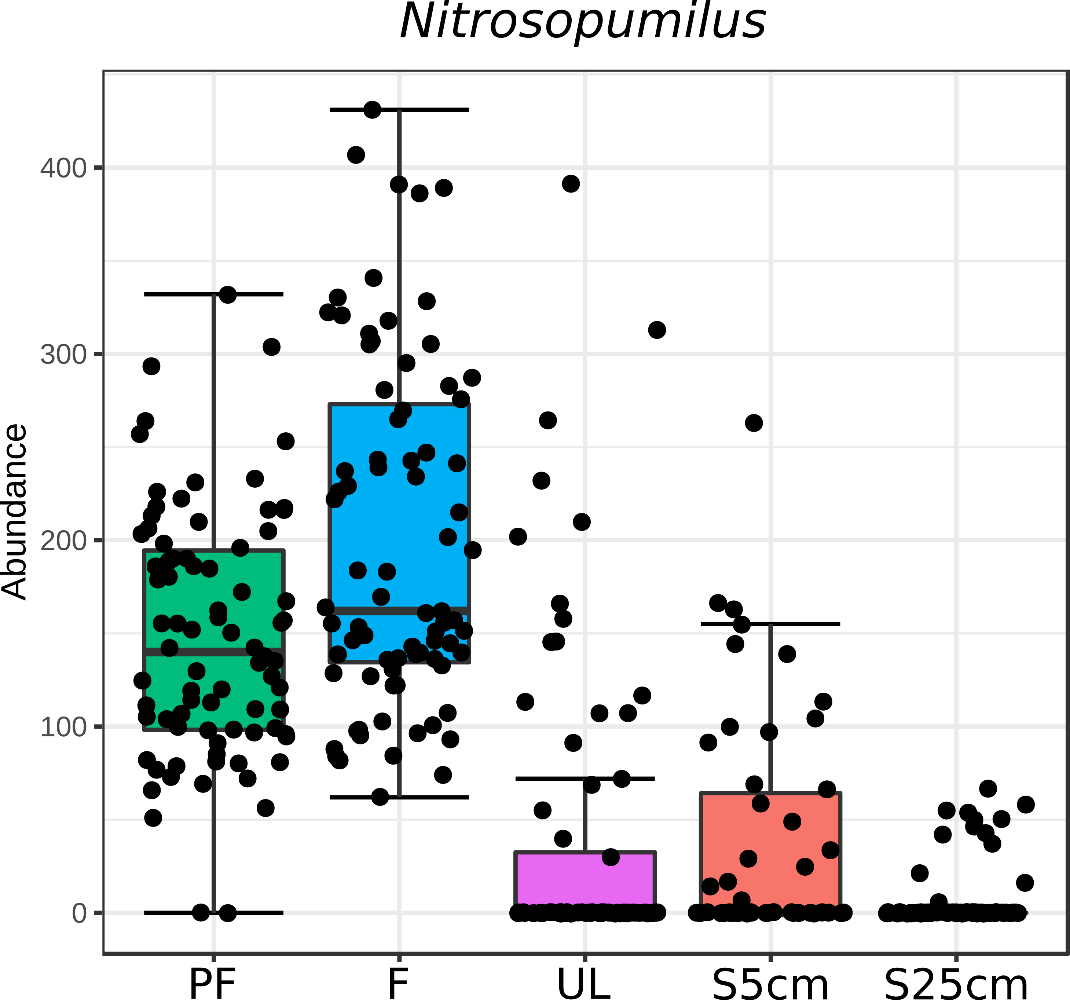


A

B


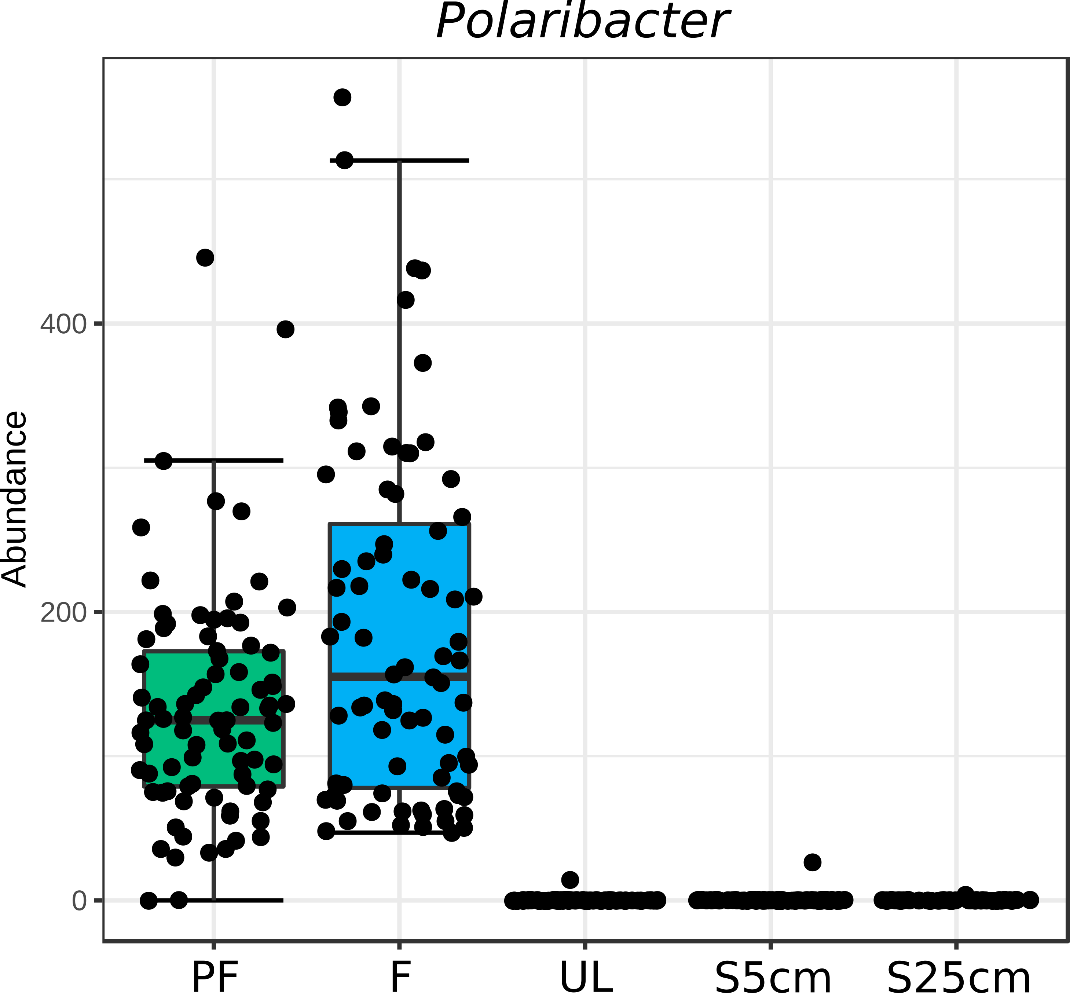


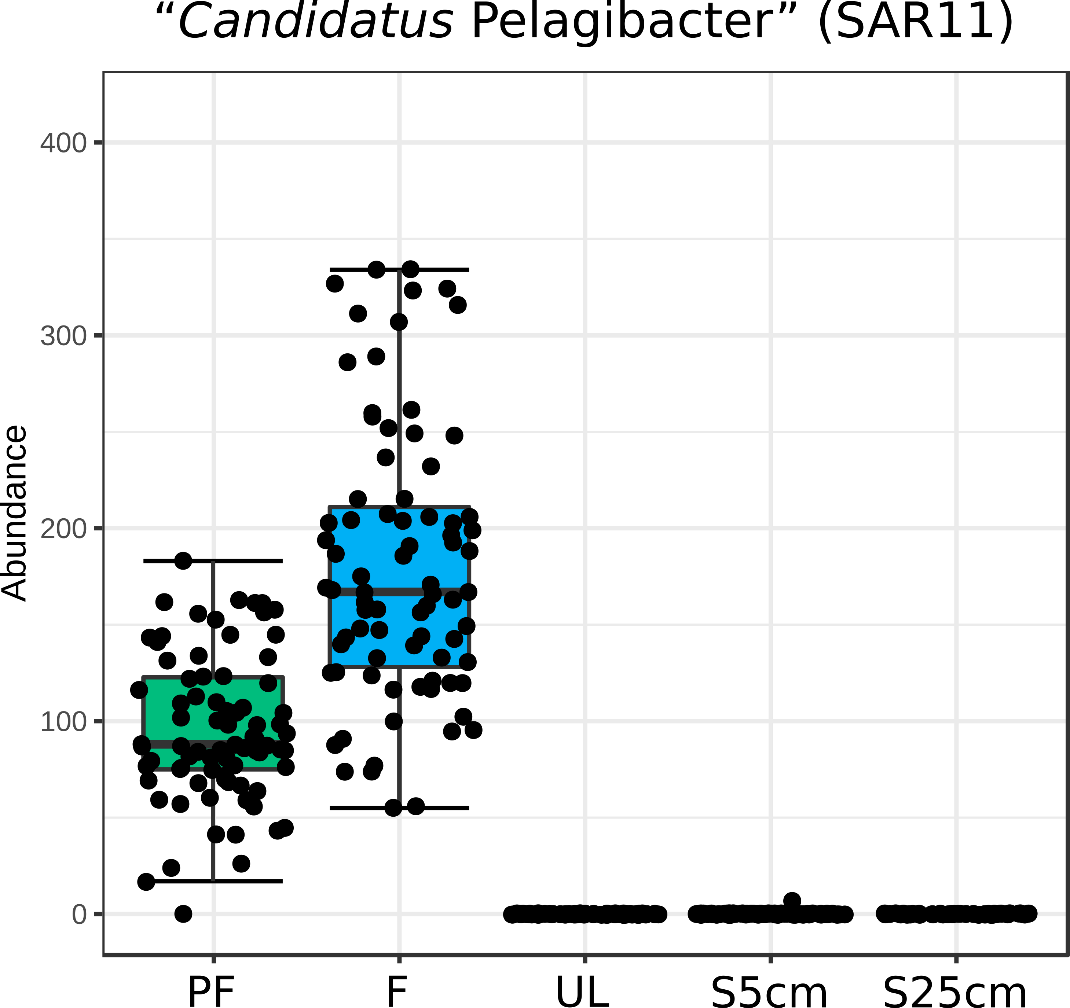


C

D


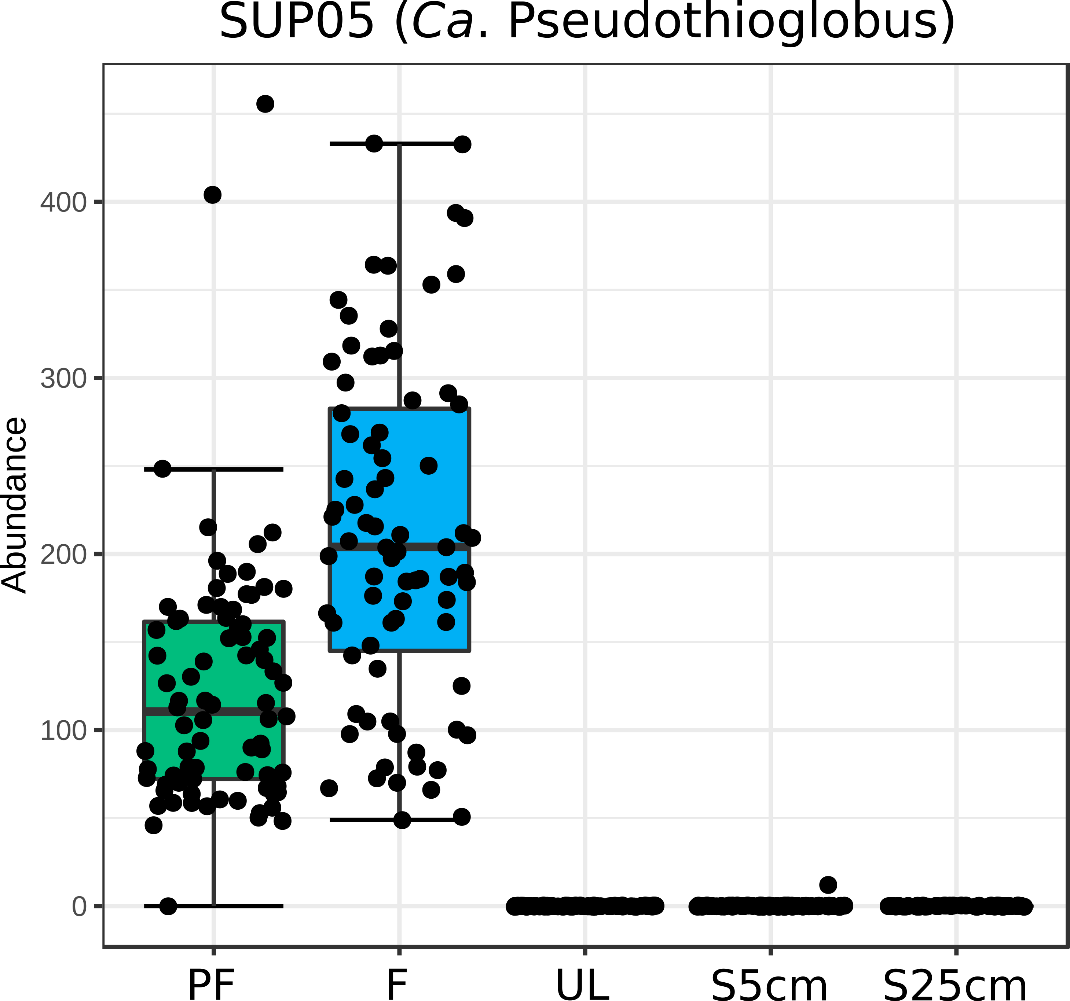


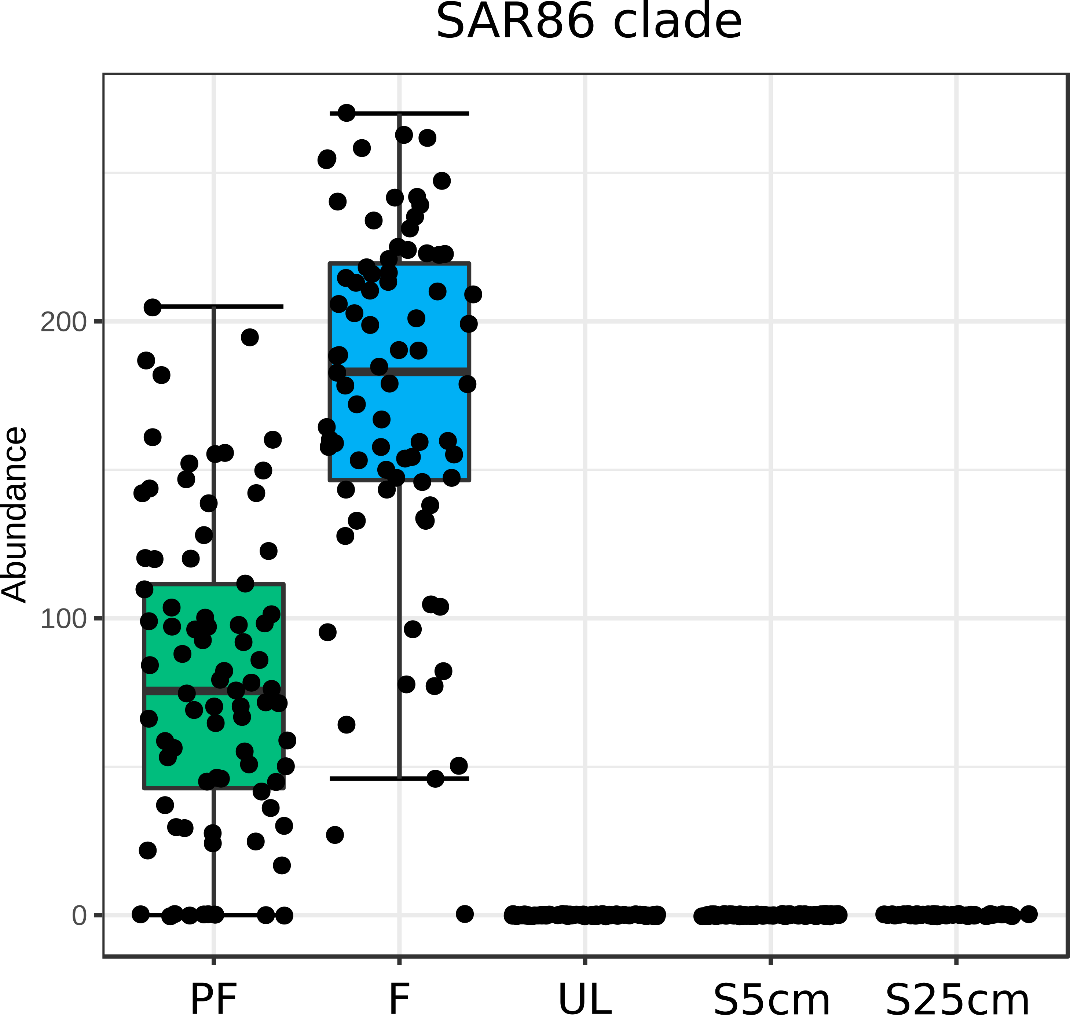


E


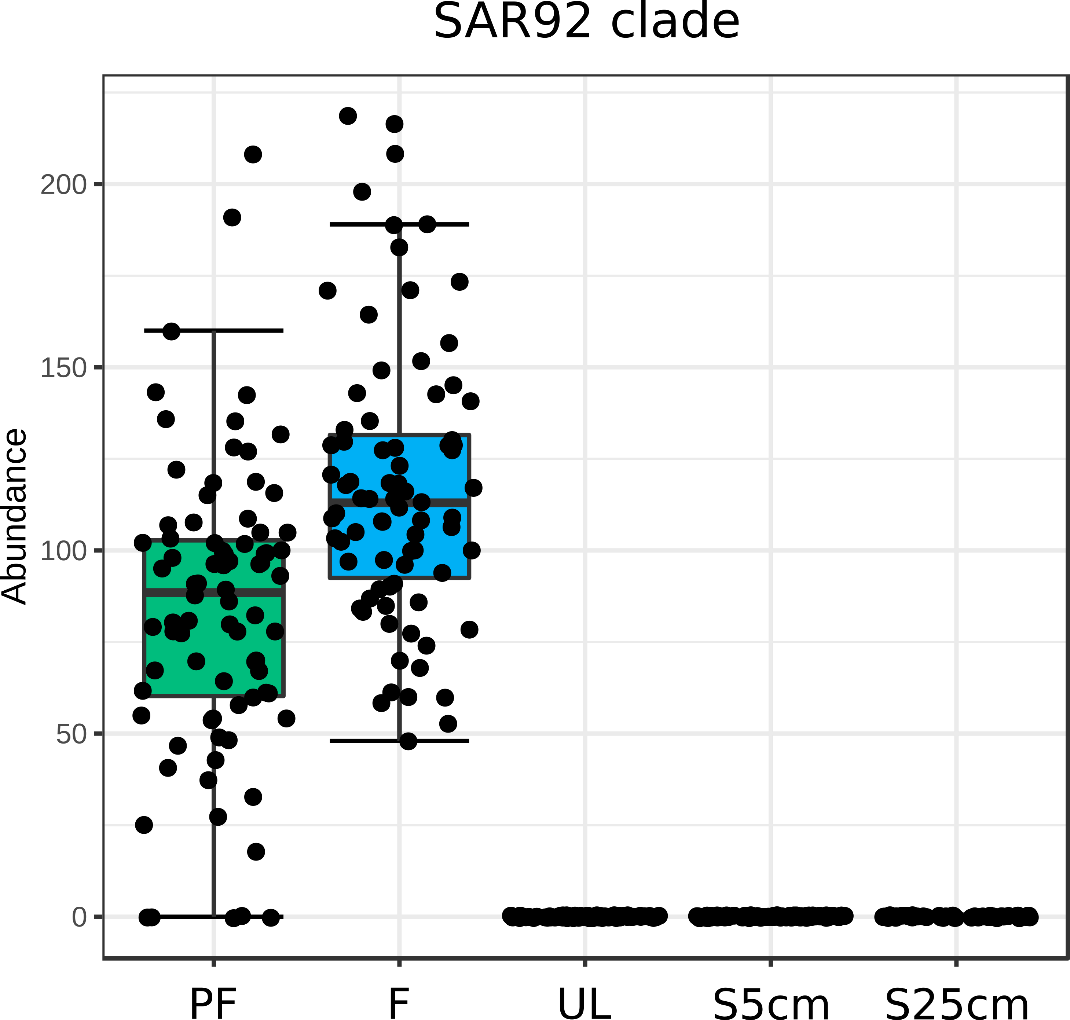


F


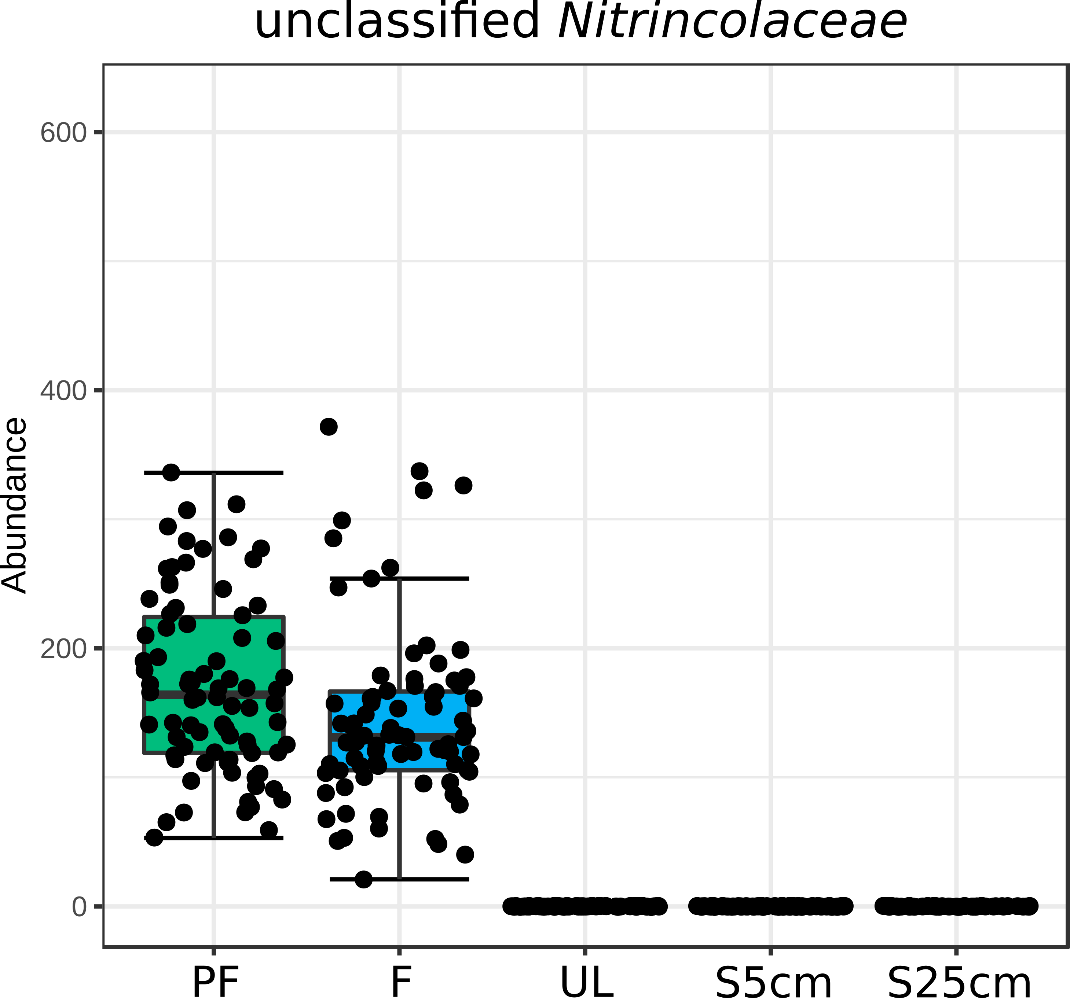


G


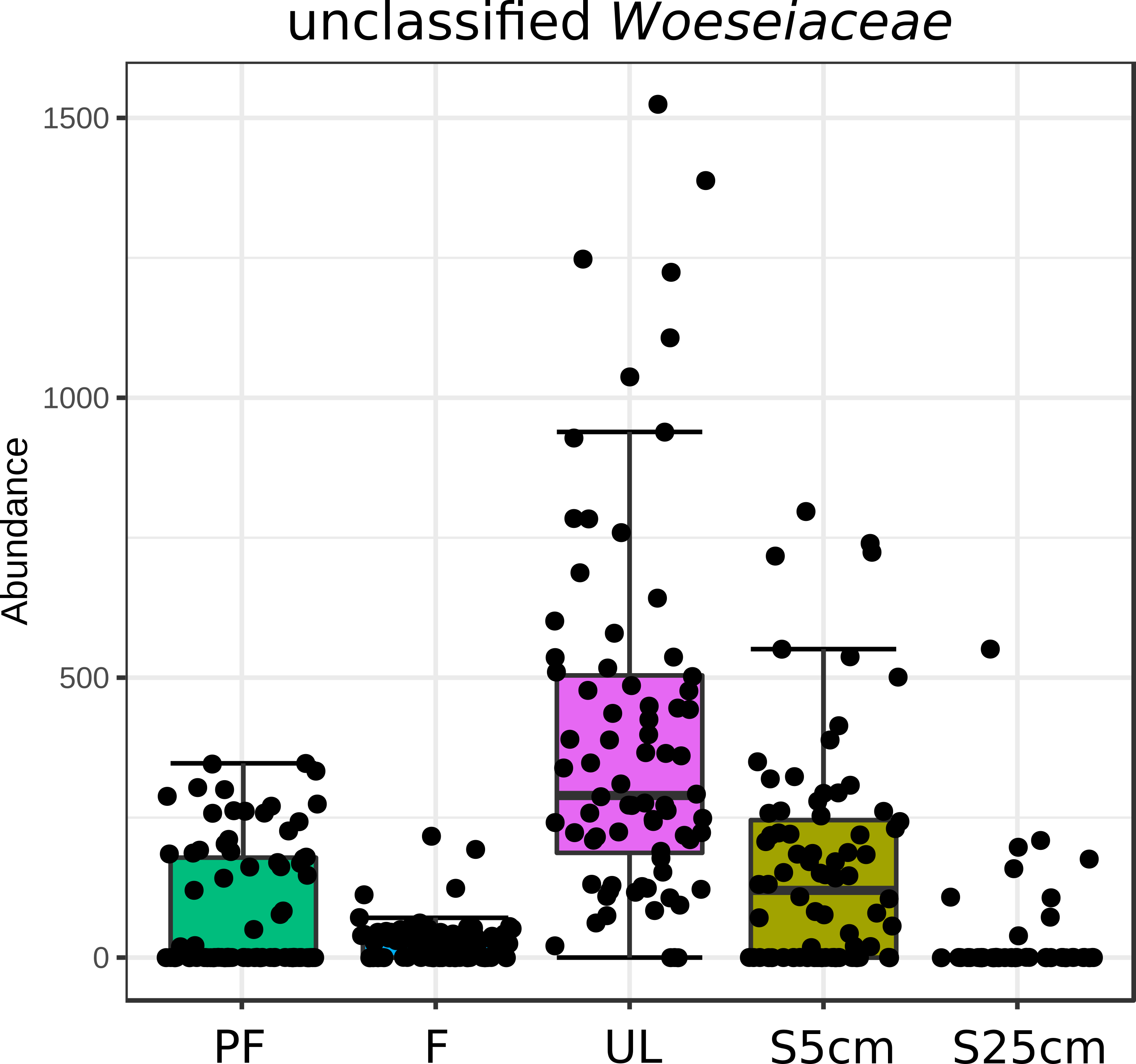


H


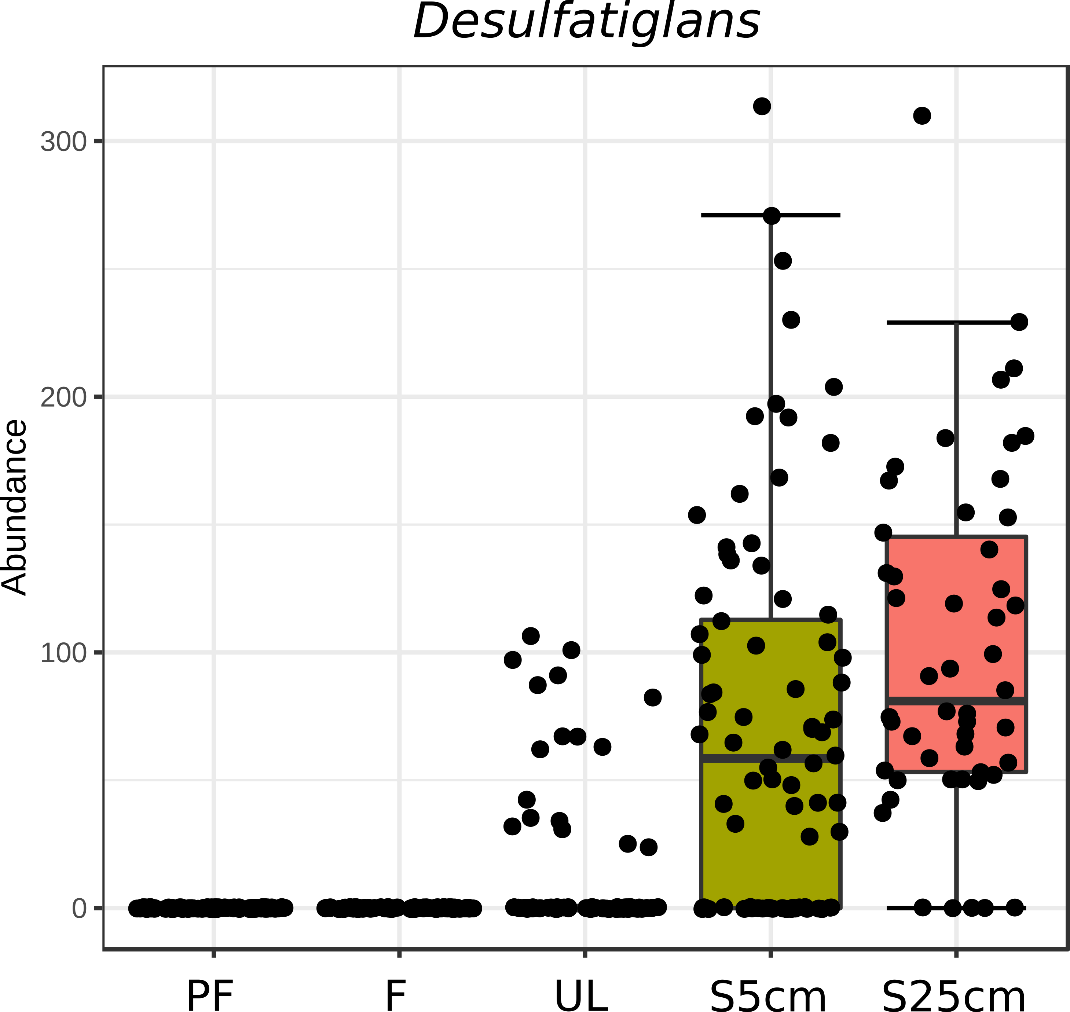


I


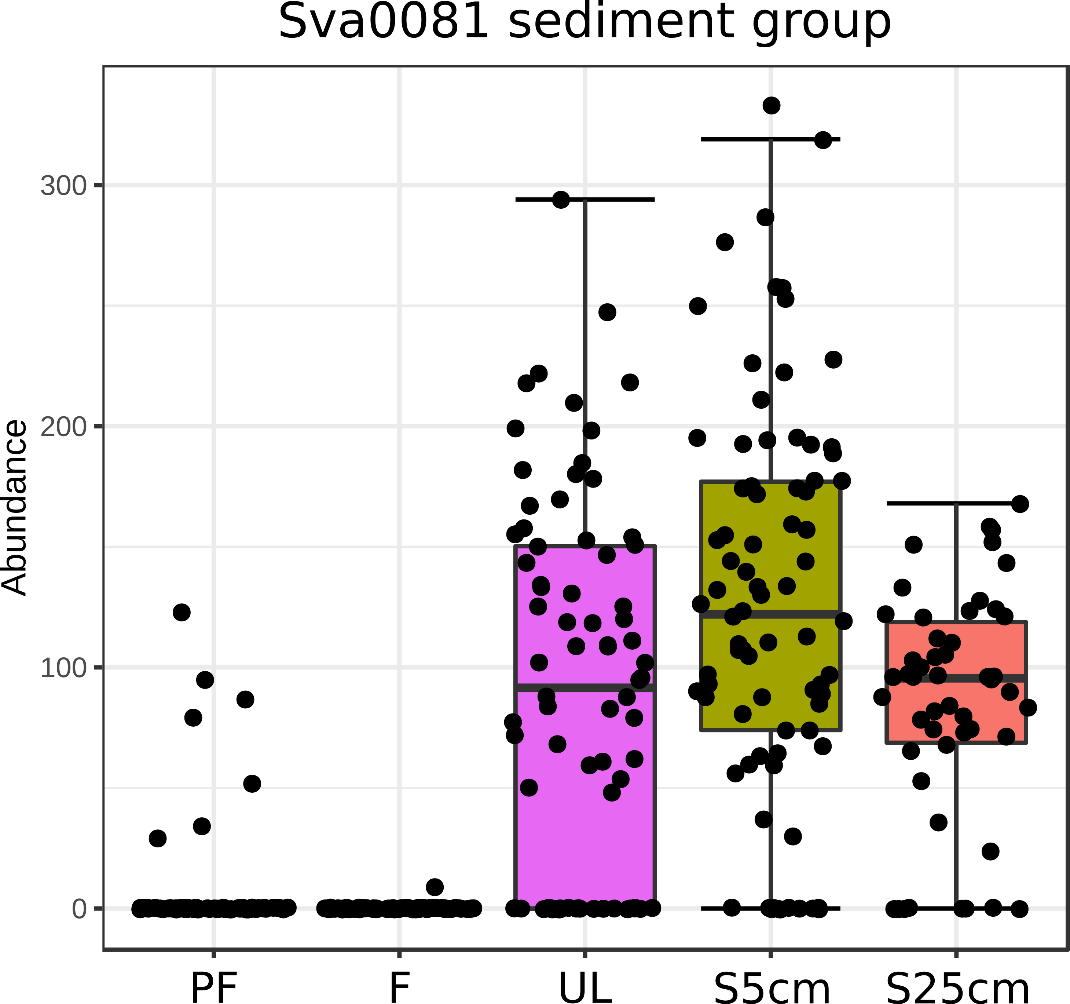


J


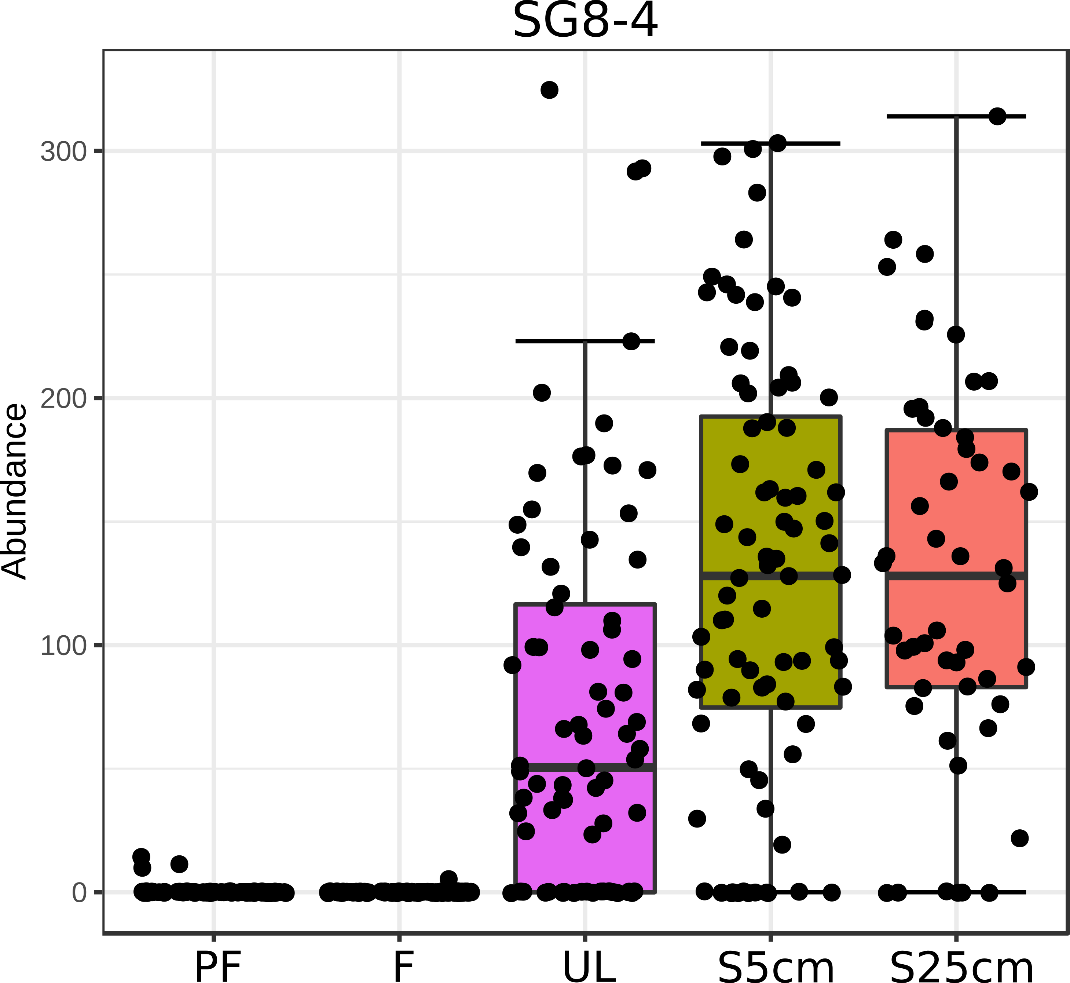


K

L


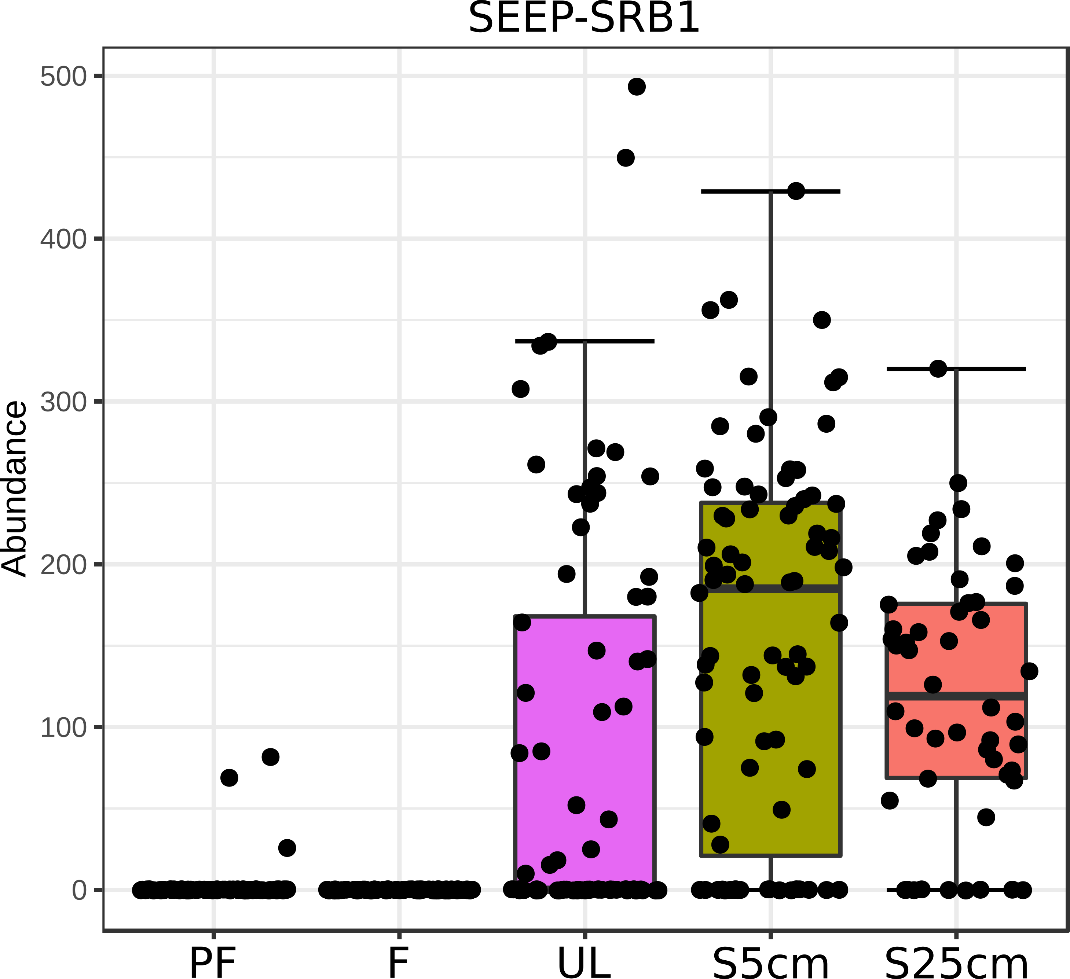


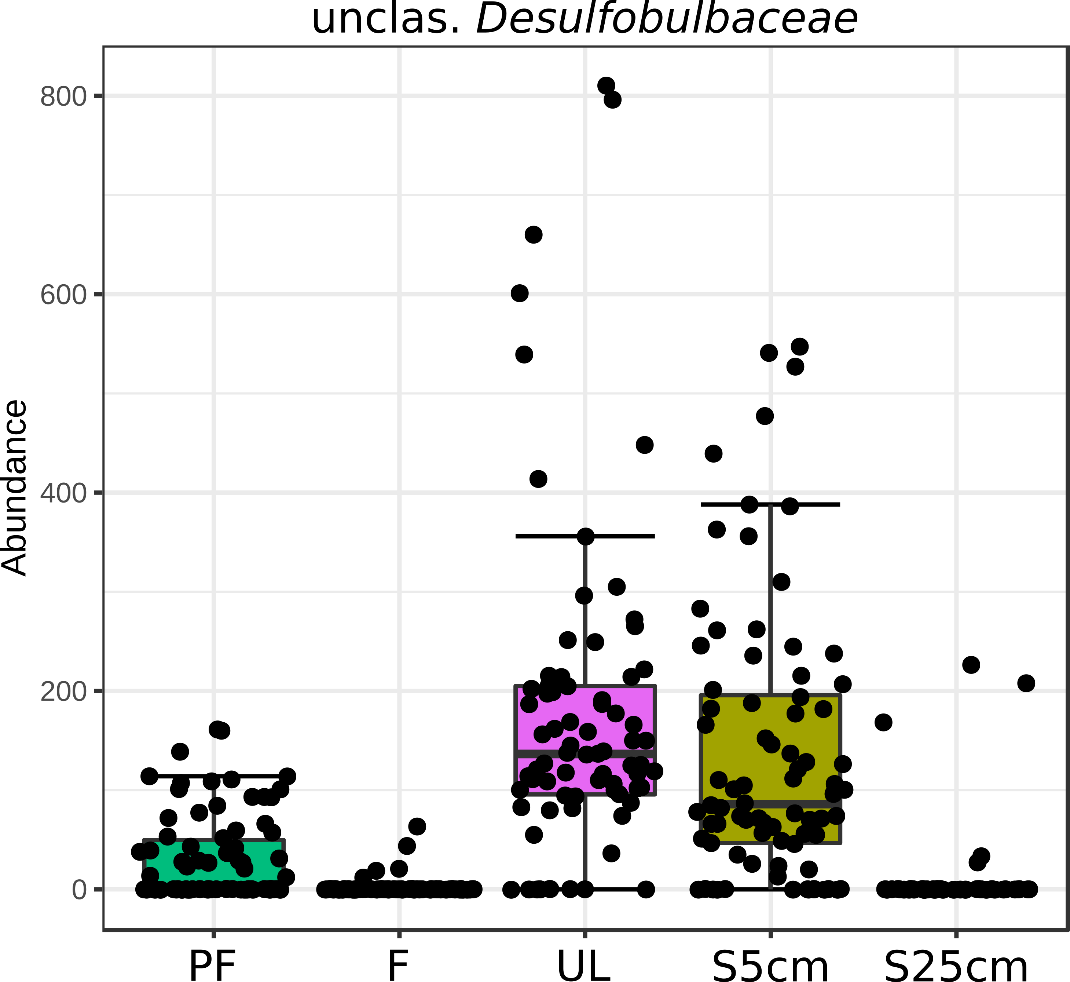


M

N


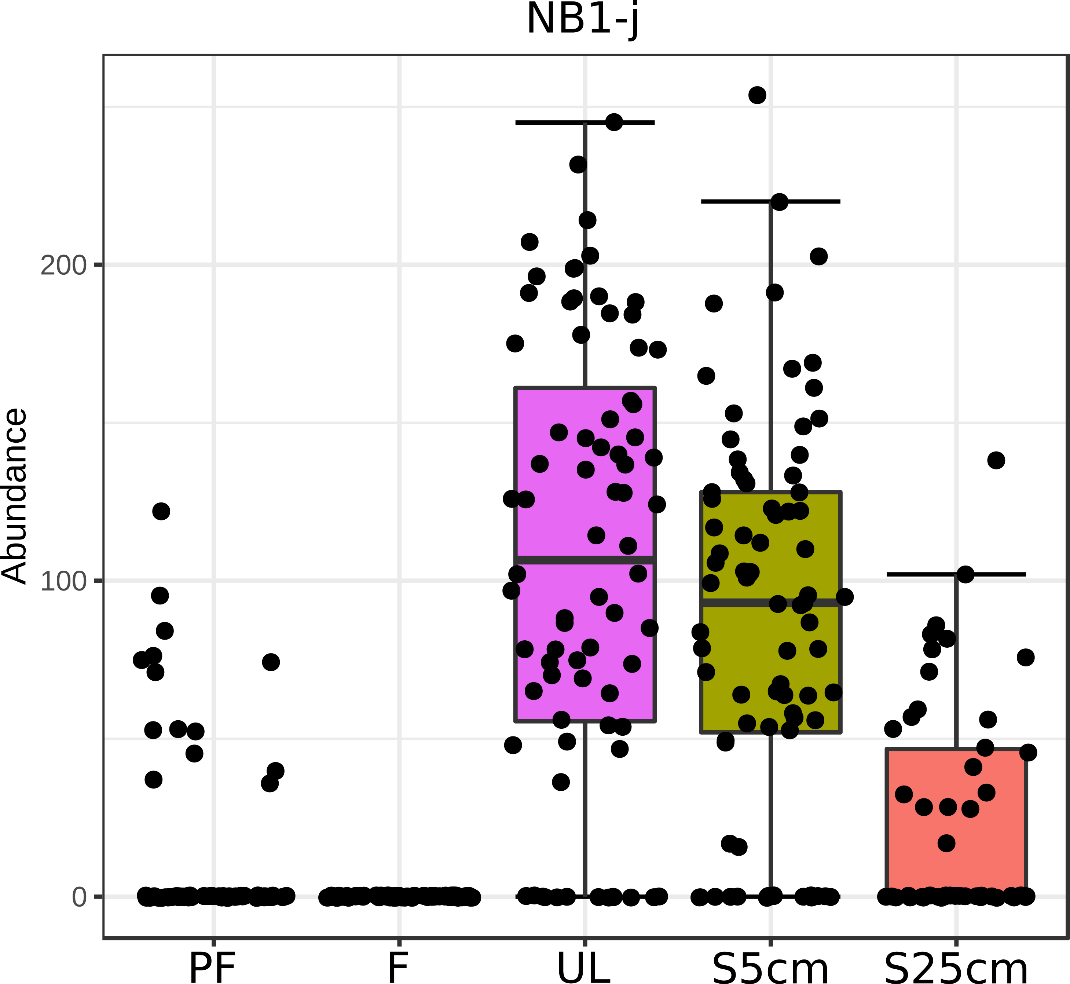


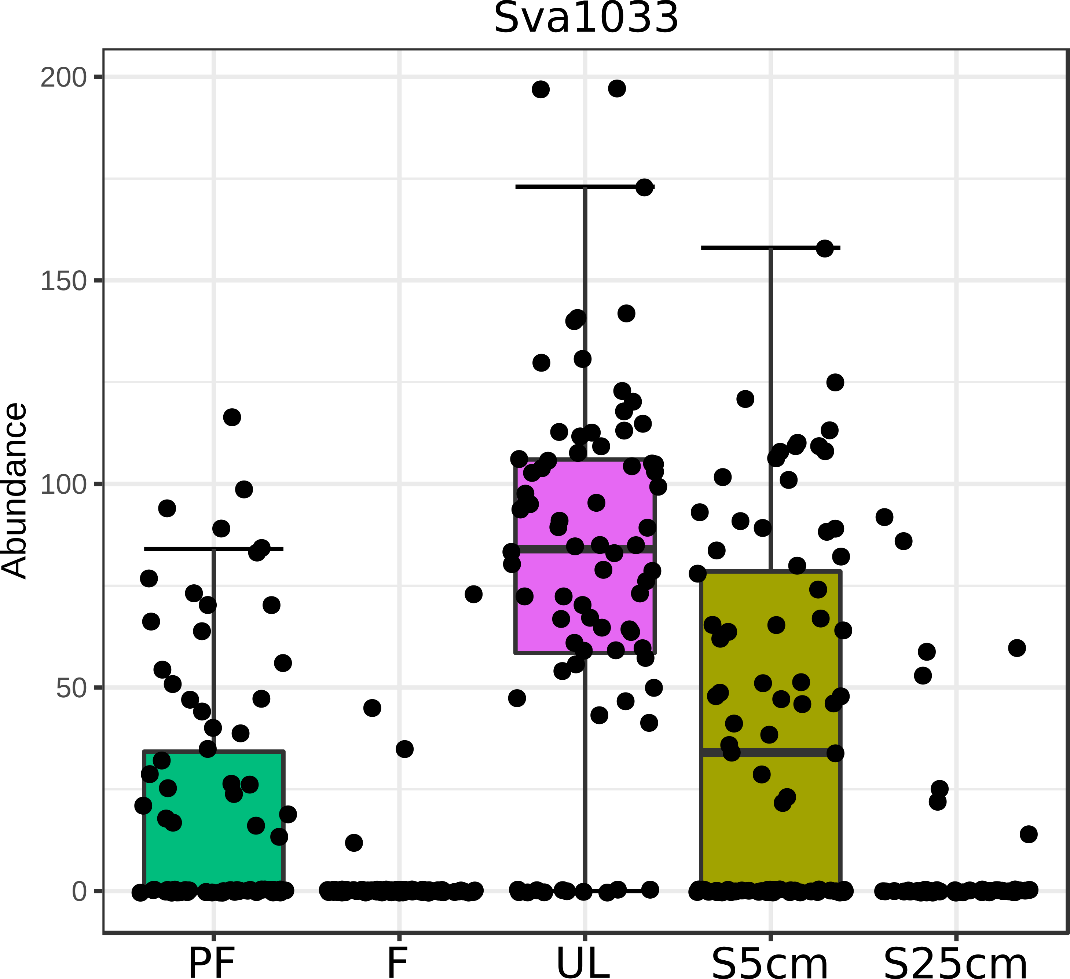


O

P


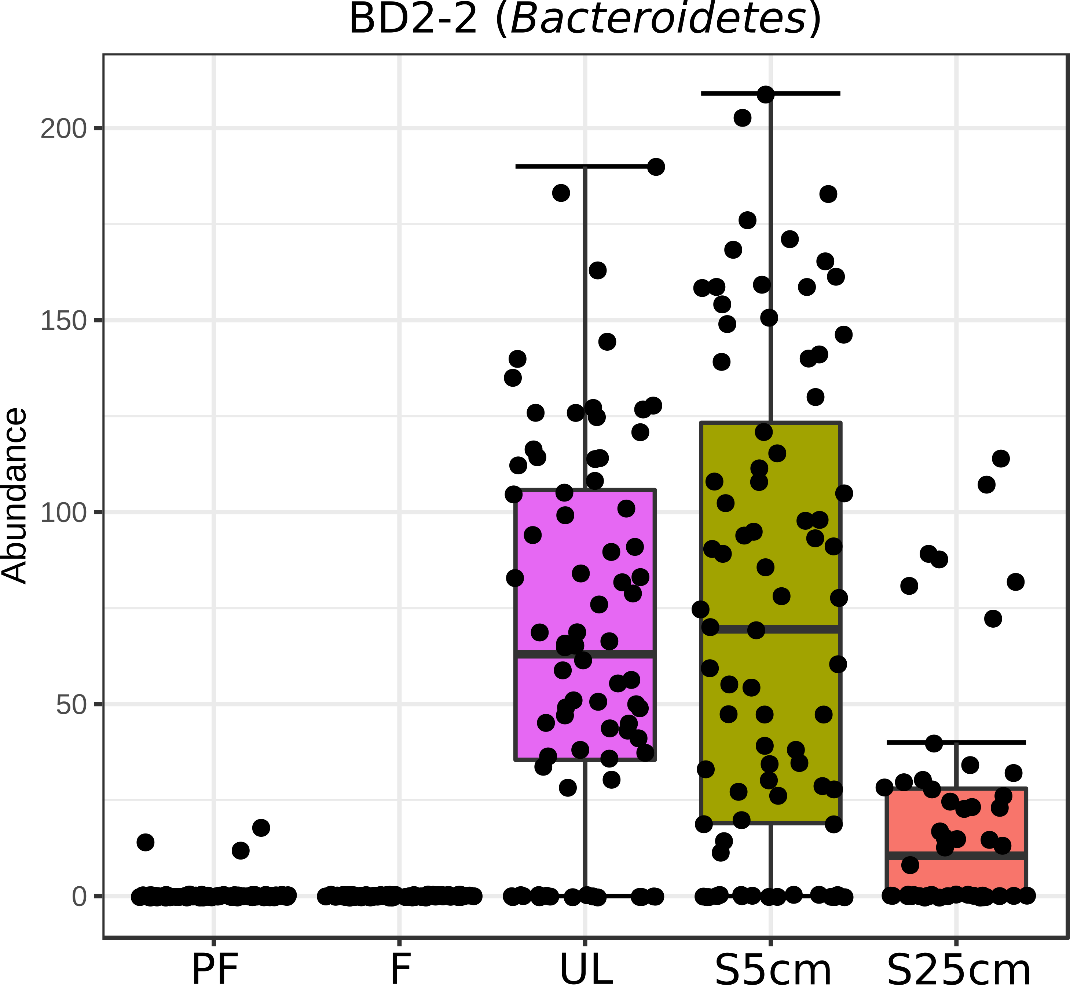


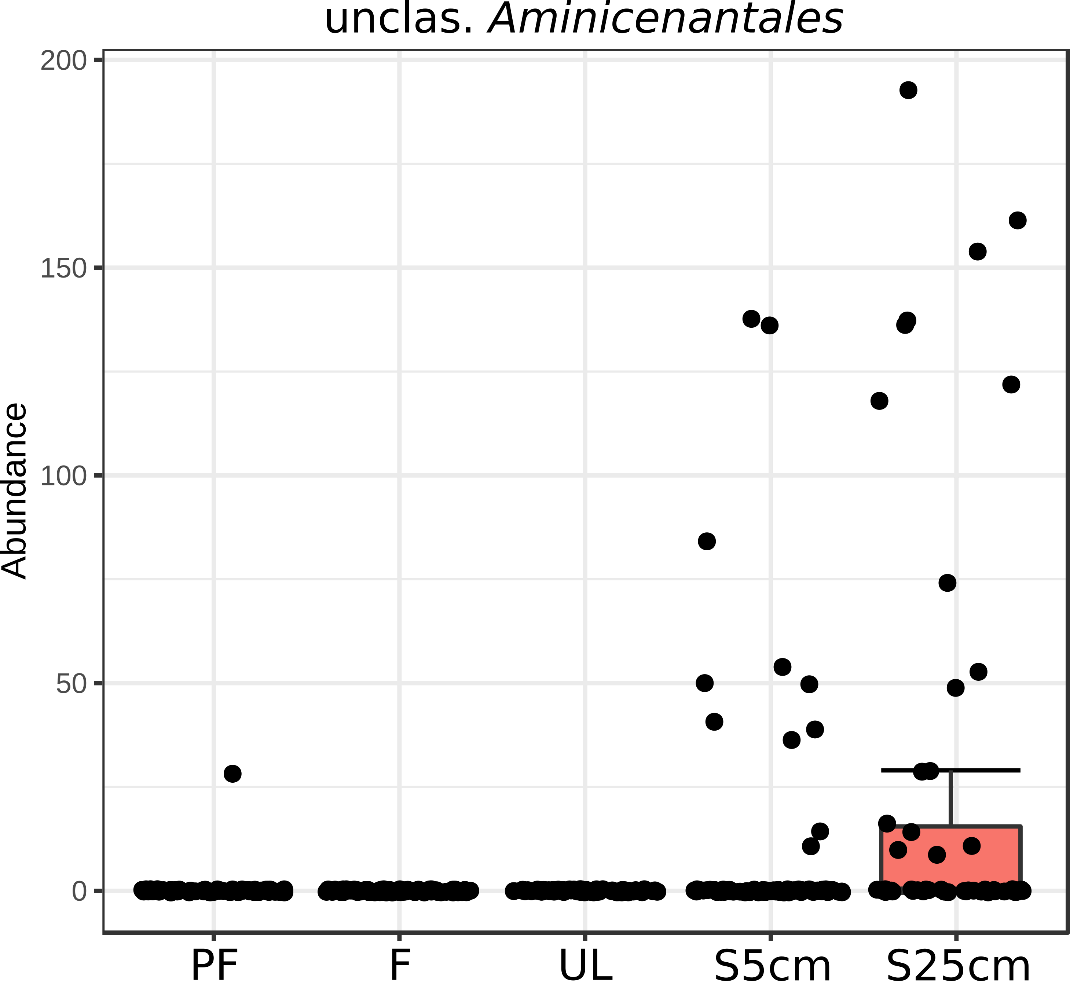


Q

R


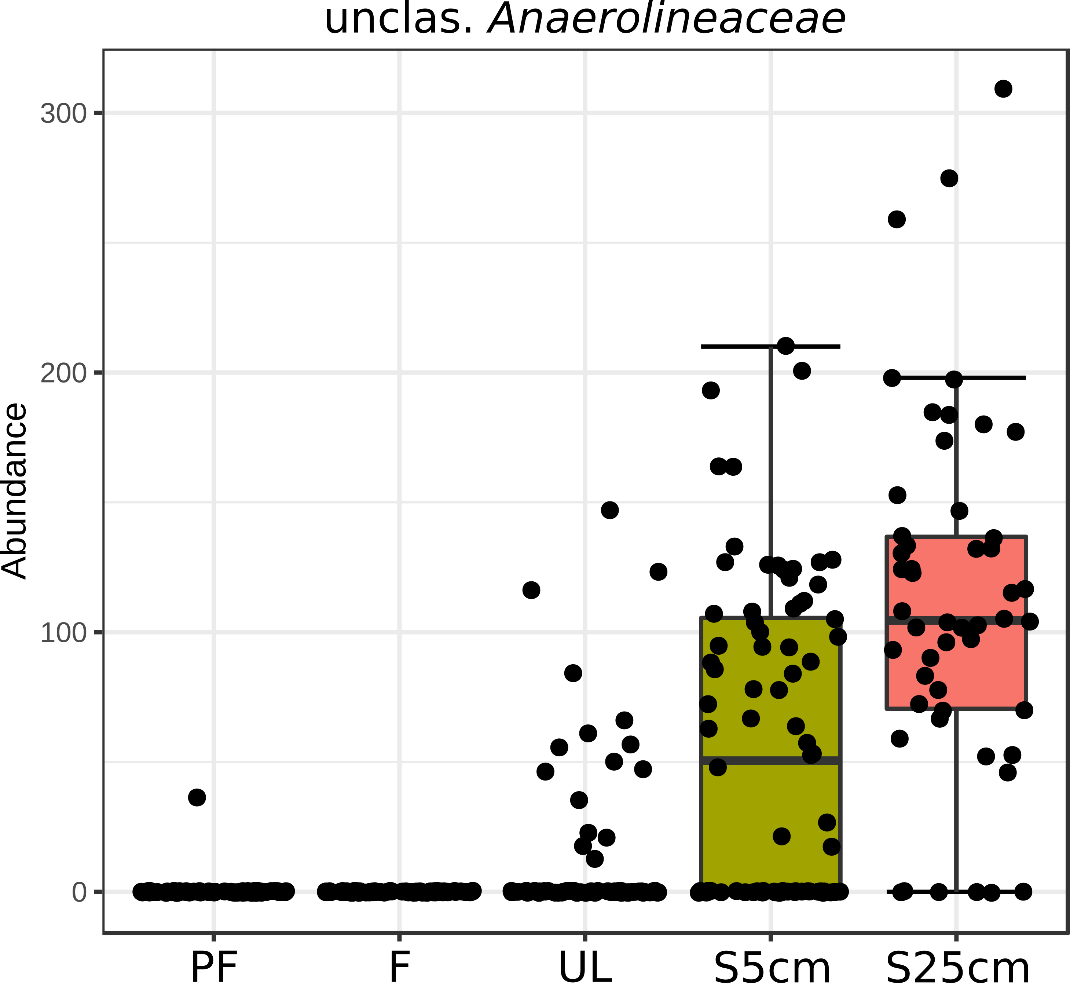


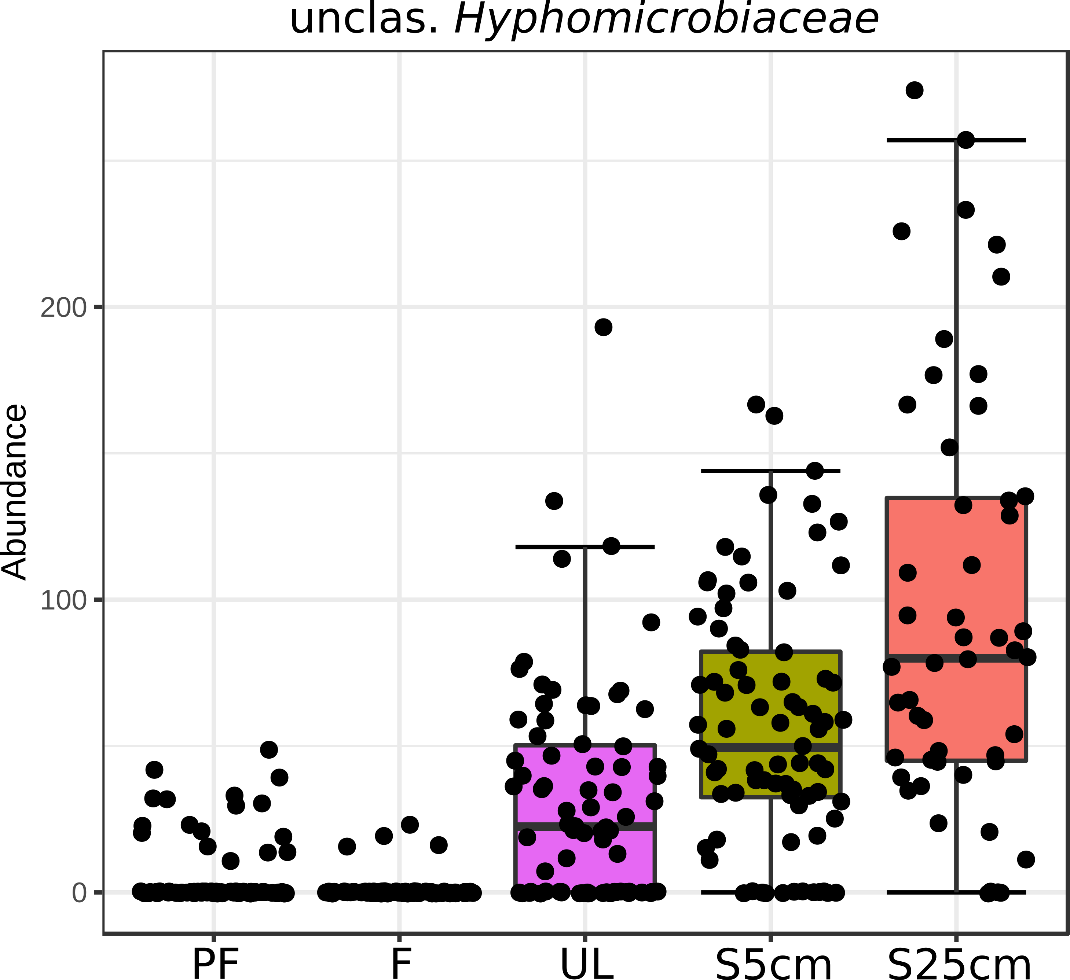


S

**FIGURE S1.** Distribution of ASV abundance of different groups in normalized data for each sample type. Sample types are 1) prefilter samples (PF), 2) filter samples (F), 3) uppermost layer samples (UL), 4) samples of sediments at 5cm depth below the bottom surface (S5cm) and 5) samples of sediments at 25cm depth below the bottom surface (S25cm). Group names are given according to SILVA SSU 138.1 (Quast et al., 2013).
